# Population-scale genomic medicine with the Hong Kong Genome Project

**DOI:** 10.1038/s41591-026-04410-w

**Published:** 2026-05-15

**Authors:** Dingge Ying, Ching-Lung Cheung, Chun-Kwan O, Wai Kei Jacky Lam, Shiu Lun Au Yeung, Chak Sing Lau, Ho Ming Luk, Christopher Kai Shun Leung, Desiree Man Sik Tse, James Si Chai Liu, Shirley Pik Ying Hue, Jamie Sui Lam Kwok, Denis Long Him Yeung, Christopher Brandon Preusch, Wei Ma, Wenshu Tang, Amy Hin Yan Tong, Lisa Wing Chi Au, Juliana Chung-Ngor Chan, Yap-Hang Chan, Shirley Sze Wing Cheng, Shuk Ching Chong, Cheuk Wing Fung, Stephanie Ho, Suhas Krishnamoorthy, Gabriel Matthew Leung, Philip Hei Li, Qing Li, Herbert Ho-Fung Loong, Rashid Nok Shun Lui, Shan Luo, Becky Mingyao Ma, Ronald Ching Wan Ma, Rong Na, Kathryn Choon Beng Tan, Sheila Suet-Na Wong, Su-Vui Lo, Hong Kong Genome Project, Dingge Ying, Desiree Man Sik Tse, James Si Chai Liu, Shirley Pik Ying Hue, Jamie Sui Lam Kwok, Denis Long Him Yeung, Christopher Brandon Preusch, Wei Ma, Wenshu Tang, Amy Hin Yan Tong, Su-Vui Lo, Annie Tsz Wai Chu, Brian Hon Yin Chung, Annie Tsz Wai Chu, Brian Hon Yin Chung

**Affiliations:** 1 Hong Kong Genome Institute, Hong Kong SAR, China; 2 Department of Pharmacology and Pharmacy, Li Ka Shing Faculty of Medicine, The University of Hong Kong, Hong Kong SAR, China; 3 Laboratory of Data Discovery for Health Limited (D24H), Hong Kong Science Parks, Hong Kong SAR, China; 4 Hinda and Arthur Marcus Institute for Aging Research, Hebrew SeniorLife, Boston, MA USA; 5 Department of Medicine and Therapeutics, Faculty of Medicine, The Chinese University of Hong Kong, Prince of Wales Hospital, Hong Kong SAR, China; 6 Li Ka Shing Institute of Health Sciences, The Chinese University of Hong Kong, Hong Kong SAR, China; 7 Department of Chemical Pathology, The Chinese University of Hong Kong, Prince of Wales Hospital, Hong Kong SAR, China; 8 School of Public Health, Li Ka Shing Faculty of Medicine, The University of Hong Kong, Hong Kong SAR, China; 9 Department of Medicine, School of Clinical Medicine, Li Ka Shing Faculty of Medicine, The University of Hong Kong, Hong Kong SAR, China; 10 Department of Clinical Genetics, Hong Kong Children’s Hospital, Hong Kong SAR, China; 11 Department of Ophthalmology, School of Clinical Medicine, Li Ka Shing Faculty of Medicine, The University of Hong Kong, Hong Kong SAR, China; 12 Department of Ophthalmology, Queen Mary Hospital, Hong Kong SAR, China; 13 Hong Kong Eye Hospital, Hong Kong SAR, China; 14 Grantham Hospital, Hong Kong SAR, China; 15 Hong Kong Institute of Diabetes and Obesity, The Chinese University of Hong Kong, Prince of Wales Hospital, Hong Kong SAR, China; 16 Department of Paediatrics, The Chinese University of Hong Kong, Prince of Wales Hospital, Hong Kong SAR, China; 17 Department of Obstetrics and Gynaecology, The Chinese University of Hong Kong, Hong Kong SAR, China; 18 Joint Baylor-CUHK Center of Medical Genetics, The Chinese University of Hong Kong, Hong Kong SAR, China; 19 Department of Paediatrics and Adolescent Medicine, Hong Kong Children’s Hospital, Hong Kong SAR, China; 20 Department of Clinical Oncology, The Chinese University of Hong Kong, Prince of Wales Hospital, Hong Kong SAR, China; 21 State Key Laboratory of Translational Oncology, The Chinese University of Hong Kong, Hong Kong SAR, China; 22 Medical Data Analytics Centre, The Chinese University of Hong Kong, Hong Kong SAR, China; 23 Institute of Digestive Disease, The Chinese University of Hong Kong, Hong Kong SAR, China; 24 Department of Family Medicine and Primary Care, School of Clinical Medicine, Li Ka Shing Faculty of Medicine, The University of Hong Kong, Hong Kong SAR, China; 25 Department of Surgery, School of Clinical Medicine, Li Ka Shing Faculty of Medicine, The University of Hong Kong, Hong Kong SAR, China; 26 Department of Paediatrics and Adolescent Medicine, Li Ka Shing Faculty of Medicine, The University of Hong Kong, Hong Kong SAR, China

**Keywords:** Health policy, Genetics research, DNA sequencing, Personalized medicine, Population genetics

## Abstract

The Hong Kong Genome Project (HKGP) aims to build a foundational resource for precision medicine in the Chinese population through large-scale genome sequencing and integrated analyses. Here we report findings from over 20,000 HKGP participants across two cohorts: a rare disease cohort including 2,227 patients with suspected genetic diseases and a population cohort including 18,261 participants undergoing genomic screening for medically actionable findings. The rare disease cohort achieved a diagnostic rate of 25%. When benchmarked against panels designed for European ancestries, the analysis revealed that 3.7% of the individuals in the population cohort had pathogenic or likely pathogenic variants associated with dominant disorders. While 48% of individuals were found to carry recessive disorder genes in the gene list based upon European ancestries, our analysis revealed that 38 additional clinically important genes would have been overlooked in the Chinese population. Pharmacogenomic analysis demonstrated that nearly all participants harbored at least one actionable phenotype, potentially informing nearly one million annual prescriptions in Hong Kong. The ongoing HKGP establishes a curated Hong Kong Chinese reference for clinically relevant genetic variation and serves as a blueprint for the implementation of precision medicine in underrepresented populations.

## Abstract (web summary)

Part of the Hong Kong Genome Project, genomic analyses of more than 20,000 participants provide information on clinically relevant variants for the Chinese population and offer insights on the implementation of genomic medicine initiatives.

## Main

With rapid genomic advances, large-scale genomic projects and global initiatives have prioritized genetic etiologies through two complementary approaches: diagnostic focused, particularly for rare diseases that affect 300 million individuals worldwide, and comprehensive precision medicine platforms^1^.

For diagnostic purposes, the 100,000 Genomes Project in the UK achieved a diagnostic yield of 25% for rare diseases prior to National Health Service (NHS) genome sequencing (GS) implementation^2^ and expanded to cancer and pharmacogenomics studies^3,4^. Australian Genomics primarily focused on evidence generation that subsequently informed in policy and practice to improve the equitable access to diagnostic testing^5^. In the United States, the All of Us Research Program exemplifies a comprehensive precision medicine approach by building a one-million-participant diverse genomic database to investigate genetic risk and enable applications, including pharmacogenomics^6^.

Regional genome projects, such as Singapore’s National Precision Medicine Program (NPM), Japan’s Initiative on Rare and Undiagnosed Diseases (IRUD) and Korea’s Genetic Diagnosis Program for Rare Disease (KGDP), discovered enrichment of genetic disease and population-specific founder variants identified through GS technologies, significantly improving rare disease diagnosis for Asian populations while enriching global genomic resources^7–9^. These efforts highlight the importance of delineating ethnicity-specific allele frequencies for genetic variants.

Despite these, Chinese populations are underrepresented in genomic research^7,10–12^. Prominent international genomic databases, such as the Genome Aggregation Database (gnomAD), are predominantly based on European populations^13,14^. Pathogenic variant enrichment in major populations can bias screening while variants more prevalent in underrepresented populations risk being overlooked or misclassified, leading to diagnostic delays, unnecessary testing and worsened health disparities. It limits the applicability of international genetic guidelines, including those of the American College of Medical Genetics and Genomics (ACMG) and the Clinical Pharmacogenetics Implementation Consortium (CPIC), to Chinese and other non-European populations^15–17^.

Led by the Hong Kong Genome Institute (HKGI), the HKGP addresses this gap by establishing a population-specific genomic resource for the Chinese population. It aims to improve the diagnosis and management of rare diseases, tumor syndromes and other diseases by collaborating with key stakeholders to integrate genomics into medical practice, foster research and build genomic capacity, thereby laying a foundation for world-class genomic research and widespread adoption of genomic medicine in Hong Kong.

The pilot phase of the HKGP on rare diseases with short-read GS, involving 520 probands, achieved a 24% diagnostic yield—similar to the UK’s 100,000 Genomes Project^18,19^. The ongoing main phase aims to include 100,000–120,000 genomes by 2030. By encompassing clinical applications of GS on diagnostics and beyond, this HKGP flagship study presents the project’s initial comprehensive pipeline and core findings. The integrated results derived from the project’s data involve key components and pursue four core aims: (1) characterizing population-wide diagnostic genomic variation in Hong Kong; (2) enabling early intervention and preventive care for asymptomatic individuals through an analysis of pathogenic or likely pathogenic (P/LP) variants in genes associated with dominant disorders, such as tumor syndromes and cardiovascular diseases; (3) informing reproductive planning through an analysis of carrier frequencies for recessive genetic disorders; and (4) optimizing treatment efficacy, reducing adverse drug reactions and improving therapeutic outcomes through pharmacogenomic profiling.

## Results

### Study cohort overview

To address the four aims outlined above, we structured our analyses around two complementary cohorts drawn from the 24,112 participants recruited and sequenced by the HKGP between July 2021 and November 2024. The diagnostic cohort (*n* = 2,227) comprises probands who had completed phenotype-guided diagnostic analysis, supporting personalized genetic diagnosis for individuals with suspected genetic conditions. The HKGP Chinese cohort (*n* = 18,261) comprises unrelated individuals of Chinese ancestry, selected through stringent relatedness and ethnicity filtering, to enable genotype-driven analyses of clinically actionable findings, including dominant disorder risks, recessive carrier burdens and pharmacogenomic variation.

### The diagnostic cohort for phenotype-guided genetic diagnosis

Of the HKGP participants, nearly 50% were probands—individuals firstly identified in their families as having a genetic condition—requiring personalized genetic diagnosis. Genetic diagnosis had been completed for 2,227 probands (904 singletons and 1,323 probands from various family structures), including the 520 probands enrolled in the pilot phase^18^, constituting the diagnostic cohort summarized in this study. The cohort had a balanced sex distribution and represented a wide range of age groups (<18 years: 37.6%; 18–60 years: 42.6%; >60 years: 19.8%). Most participants were Chinese (95.0%), with the minority being mixed Chinese or other ethnicities (Extended Data Table 1).

### Determinants of diagnostic yields

**Fig. 1:**
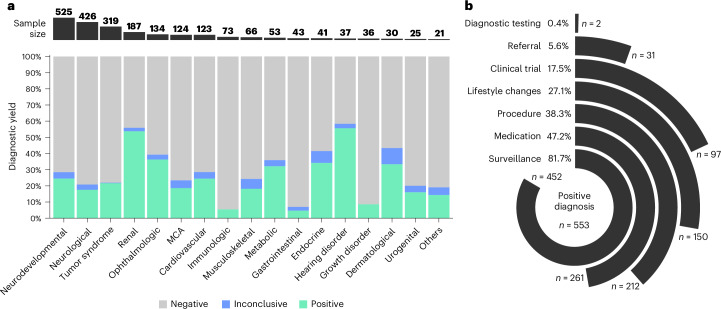
Summary of the findings from the diagnostic cohort (*n* = 2,227). **a**, The upper panel shows the proband sample size for each disease category. The lower bar chart shows the percentages of positive (green), inconclusive (blue) and negative (gray) diagnoses across 17 disease categories. The diagnostic yields varied across the disease categories, with an average of 24.8%. **b**, The circle bar chart shows the number and percentage of probands whose positive genetic diagnosis brings potential changes to clinical management as follows: 452 (81.7%) were recommended for enhanced surveillance, such as increased cancer screening or disease monitoring; 261 (47.2%) received guidance on medication use, including avoidance of adverse drug reactions or optimized dosing; 212 (38.3%) were provided with indications or contraindications for specific medical procedures, identifying contraindications for surgeries or indicating preventive interventions; and 150 (27.1%) would benefit from lifestyle modifications, such as dietary changes or activity restrictions. Among the 97 (17.5%) probands eligible for clinical trials, 75 (13.6%) were eligible for interventional clinical trials, with nine of these involving phase 3/4 trials, whereas 22 were eligible only for observational clinical trials. Notably, *GJB2*-related hearing loss and *NF1*-related neurofibromatosis account for 50 of the 97 probands. Most probands (488, 88.2%) could benefit from multiple categories of clinical management, mostly guided by recommendations related to surveillance, medication and procedures. MCA, multiple congenital anomalies. Source data

Comprehensive variant detection and curation identified positive genetic diagnoses for 553 out of the 2,227 probands (24.8%), consistent with the pilot phase^18^. The diagnostic yields varied across disease categories (4.65−56.8%; Fig. 1a). Thirteen probands received multiple diagnosis, with P/LP variants in two genes explaining distinct phenotypes in the same individual (Supplementary Table 1). Subgroup analyses with *χ*^2^ test revealed that non-singleton probands presented a higher yield (26.4%) than singletons (22.6%) (*P* = 0.041), highlighting the value of sequencing family members to enhance variant interpretation. The subgroup with more than eight Human Phenotype Ontology (HPO) terms had a significantly higher yield (27.1%) than that with fewer terms (22.6%) (*P* = 0.014). The diagnostic yields were slightly higher in adult probands and in those with previous genetic testing (Extended Data Table 2).

### Variants in the diagnostic cohort

Among the 553 probands with positive genetic diagnoses, a total of 572 unique P/LP variants were identified through ACMG guideline-based curation. Of these, 486 (85.0%) were single-nucleotide variants (SNVs) or small insertions and deletions (indels) across 350 genes, whereas 86 (15.0%) were copy number variants (CNVs), structural variants (SVs) or short tandem repeats (STRs), spanning 30 genes. Among the SNVs/indels, nonsense (22.4%), frameshift (22.4%) and missense (41.6%) were common. The CNVs/SVs/STRs were predominantly deletions (58.1%) and repeat expansions (19.8%).

Of the identified P/LP SNVs/indels, 31.1% were novel, and 68.9% had been previously reported in ClinVar. Sixty-two (12.7%) variants labeled as uncertain significance or conflicting pathogenicity in ClinVar were reclassified as P/LP. Of the CNV/SV/STR variants, 57.6% were novel, and 42.4% had been reported in ClinVar, in GeneReviews (www.ncbi.nlm.nih.gov/books/NBK1116/) or in other literature (Table 1 and Supplementary Table 2) as P/LP^20^.

**Table 1:** Summary of variant classifications for different variant types in the HKGP

Mutation droup	Type	Diagnostic cohort (probands with positive finding, *n* = 553)			HKGP Chinese cohort (ACMG SF dominant genes, *n* = 73)			HKGP Chinese cohort (ACMG CS recessive genes, *n* = 105)	
		Reported P/LP^c^	Reclassified P/LP	Novel P/LP (FM)	Reported P/LP	Reclassified P/LP	Novel P/LP (FM)	Reported P/LP	Novel P/LP (FM)
SNV/indel	Frameshift	45	3	61 (1)	61	2	50	209	8 (1)
	Nonsense	80	2	27	52	13	27	226	7
	Missense	116	52	34 (1)	60	48	11	516	0
	Splice	21	4	14	19	11	5	147	3
	Inframe indel	1	0	4	0	1	0	16	0
	Other^a^	10	1	11	1	0	0	38	0
	Total	273	62	151	193	75	93	1152	18
	Total (%)	56.2	12.7	31.1	53.4	20.8	25.8	98.5	1.5
CNV/SV/STR	Deletion	12	0	38	2	0	8	2	46
	Duplication	3	0	3	0	0	1	1	13 (1)
	Insertion	2	0	1	0	0	0	1	0
	Other^b^	3	0	7	0	0	1 (1)	0	0
	Repeat expansion	17	0	0	0	0	0	0	1
	Total	37	0	49	2	0	10	4	61
	Total (%)	43.0	0.0	57.0	16.7	0.0	83.3	6.2	93.8

^a^ Includes intronic, complex, synonymous, tRNA-coding and 5′ untranslated region (UTR) variants

^b^ Includes variants combined by indels with inversions, translocations and other types of CNV/SV mentioned in the table

^c^ Reported in public resources (ClinVar, GeneReviews) or publications

CS, carrier screening gene list—both autosomal and X-linked genes were included; FM, founder mutation; SF, secondary finding, ACMG version 3.2 gene list.

### Potential clinical management

Among the probands with a positive diagnosis in the diagnostic cohort, GS ended the average diagnostic odyssey of 13 years. To assess the clinical utility of the diagnoses, we classified the potential changes in clinical management into seven categories. GS provided diagnoses that altered the clinical trajectory of 488 (88.2%) probands, reducing the diagnostic burden on probands and families through potential clinical management. Specifically, the minimal need for additional testing (two probands, 0.367%) confirmed GS as the penultimate diagnostic tool (Fig. 1b and Supplementary Table 2).

### A Chinese-specific reference cohort for clinically actionable findings

The HKGP Chinese cohort includes 11,362 asymptomatic and symptomatic singletons (partially overlapped with the diagnostic cohort) and 6,899 parents. The demographic and clinical characteristics of this cohort were relatively consistent with the diagnostic cohort, with intentional differences in health status and ethnicity by design (Extended Data Table 1). The HKGP Chinese cohort not only supports genotype-driven analyses of clinically actionable variants but also represents the allele frequency landscape of the Hong Kong Chinese population.

### Variants in dominant disorder-related genes

Using the HKGP Chinese cohort, we investigated 73 dominant disorder (autosomal and X-linked) ACMG secondary finding genes (version 3.2)^16^ to assess GS utility beyond the primary indication for testing. We excluded participants with related phenotypes, resulting in 17,949 participants analyzed. Among these, 670 individuals (3.73%) carried at least one P/LP variant across 54 genes, with 20 participants carrying two or more (Supplementary Tables 3 and 4).

**Fig. 2:**
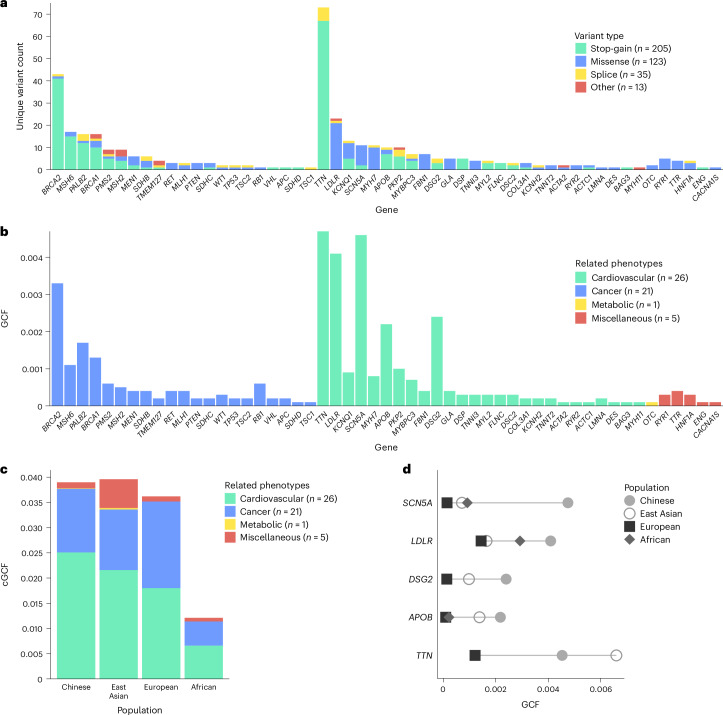
Summary of the findings in dominant genes in the HKGP Chinese cohort (*n* = 17,949). **a**, Composition of P/LP variant types across 53 of 73 dominant disorder-related genes. Stop-gain variants (frameshift or nonsense) were the most common, followed by missense and splice variants. **b**, GCF of each dominant disorder-related gene. Cancer and cardiovascular genes presented relatively high GCFs, with the frequencies of 10 genes (for example, *BRCA2*, *PALB2*, *SCN5A*, *LDLR* and *TTN*) exceeding 0.001 in the HKGP Chinese cohort. **c**, The cGCF of cardiovascular-related dominant disorder, cancer, metabolic disorder and miscellaneous genes in the HKGP Chinese cohort and in East Asian (Koreans from KoGES), European (deCODE Genetics in Iceland) and African (from All of Us project) populations. **d**, Comparison of GCFs of five autosomal dominant cardiovascular genes (*SCN5A*, *TTN*, *LDLR*, *DSG2* and *APOB*) enriched in the HKGP Chinese cohort (Chinese), comparing with East Asian, European and African populations. The enrichment reveals unique trends in variant distribution. Of note, GCFs of *DSG2* and *TTN* are not reported for African from the All of Us project. KoGES, Korean Genome and Epidemiology Study. Source data

**Extended Data Fig. 1:**
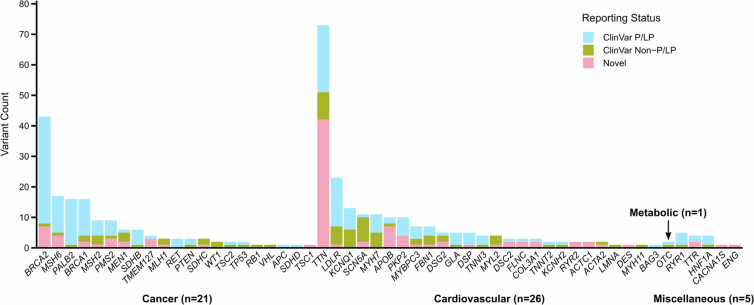
Breakdown of the reporting status of the P/LP variants for 53 of 73 dominant disorder-related genes by related phenotypes. All pathogenic or likely pathogenic (P/LP) variants, classified using the method described in this study, in dominant disorder-associated genes from the HKGP Chinese cohort were cross-referenced with the ClinVar database. Variants not reported in ClinVar were classified as novel. Variants reported in ClinVar and classified as pathogenic or likely pathogenic were labeled as ClinVar P/LP, while all others were labeled as ClinVar non-P/LP. P/LP, pathogenic or likely pathogenic. Source data

A total of 373 unique P/LP variants were identified in this analysis (Table 1). *BRCA2* and *TTN* were enriched predominantly in nonsense and frameshift P/LP variants, whereas *LDLR*, *SCN5A* and *MYH7* were enriched in missense P/LP variants (Fig. 2a). Among the 361 identified P/LP SNVs/indels, 25.8% were novel, whereas 74.2% had been previously reported in ClinVar. Seventy-five (20.8%) ClinVar non-P/LP variants were reclassified as P/LP in this study following the ACMG guidelines. Specifically, 25.3% were null variants meeting PVS1 by manual verification^21^; 64.0% were missense variants supported by high prediction scores (PP3_Strong)^22^; and 10.7% were upgraded via additional evidence from the literature or databases (Supplementary Table 5 and Extended Data Fig. 1).

### Variant prevalence in dominant disorder-related genes

To quantify the population burden of these clinically actionable variants, we calculated the gene carrier frequency (GCF) for each ACMG secondary finding gene among the 17,949 participants^23^, reflecting the prevalence of individuals carrying at least one P/LP transmissible variant in a gene regardless of their own symptomatic status. Consistent with reports from other populations, cancer-related genes (*BRCA2*, *PALB2*, *BRCA1* and *MSH6*) had high GCFs. Cardiovascular genes (*SCN5A*, *TTN*, *LDLR*, *DSG2* and *APOB*) presented GCFs markedly higher than in other continental populations^24–26^. Overall, the cumulative gene carrier frequency (cGCF) for cardiovascular genes (2.48%) was higher than cancer-related genes (1.23%) and others (0.18%) (Fig. 2b and Supplementary Table 6); slightly higher than that in another East Asian population (Korea, 2.17%); and markedly higher than that in European (Icelandic, 1.80%) and African (US based, 0.66%) populations (Fig. 2c). Notably, the enrichment of P/LP variants in *SCN5A* (long QT syndrome type 3, Brugada syndrome and dilated cardiomyopathy) and *DSG2* (arrhythmogenic right ventricular cardiomyopathy) in our cohort was not previously reported (Fig. 2d). These differences in Chinese populations can guide gene prioritization for early disease risk detection panels designed for this population.

### Carrier burden in recessive disorder-related genes

We evaluated carrier burden from autosomal and X-linked recessive genes using the ACMG pan-ethnic tier 1−3 carrier screening (CS) gene list (105 genes)^15,27^. This tiered framework—tier 1 covering universally recommended conditions; tier 2, carrier frequency ≥1/100 and moderate to severe phenotypes; and tier 3, carrier frequency ≥1/200, including autosomal recessive and X-linked genes—was developed to guide equitable and comprehensive genetic screening across diverse populations, regardless of ancestry. Among 18,065 participants whose children did not have any primary indication phenotypes linked to these genes, 8,693 individuals (48.1%) carried at least one P/LP variant in 105 ACMG CS genes (Extended Data Table 3 and Supplementary Table 7). Across these ACMG CS genes, we identified 1,235 unique P/LP variants spanning 98 genes, of which 1,170 (94.7%) were SNVs or indels, and the remaining 65 variants (5.26%) were CNVs/SVs/STRs (Table 1 and Supplementary Tables 3 and 4).

**Extended Data Fig. 2:**
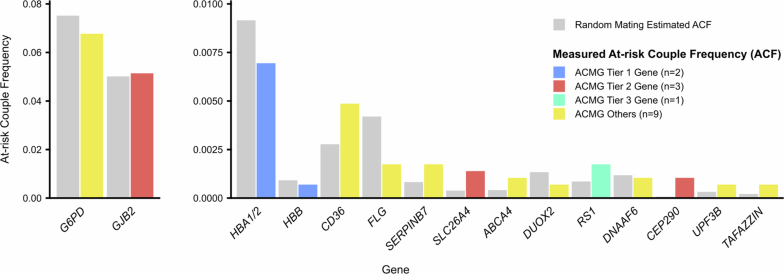
Comparison of the measured ACF and random mating estimates across the top ACF genes. A couple is at-risk if they both have P/LP variants in the same recessive disorder genes. Measured at-risk couple frequencies are highlighted by colours based on the tiers of the ACMG pan-ethnic carrier screening panel. The random mating-estimated ACF was closely aligned with the measured ACF. ACF, at-risk couple frequency. Source data

To translate carrier frequencies into reproductive risk, we estimated the at-risk couple frequency (ACF)^23^. Using a random mating approach based on these participants’ P/LP carrier status^7^, we modeled 163 million theoretical pairings. The virtual cumulative at-risk couple frequency (cACF) for ACMG CS genes was 6.60%, dominated by *GJB2* (5.01%). Validation in 2,864 actual couples yielded a cACF of 6.77% (also dominated by *GJB2*, 5.17%), closely aligned with the virtual cACF, confirming the reliability of our modeling (Supplementary Table 7 and Extended Data Fig. 2).

**Fig. 3:**
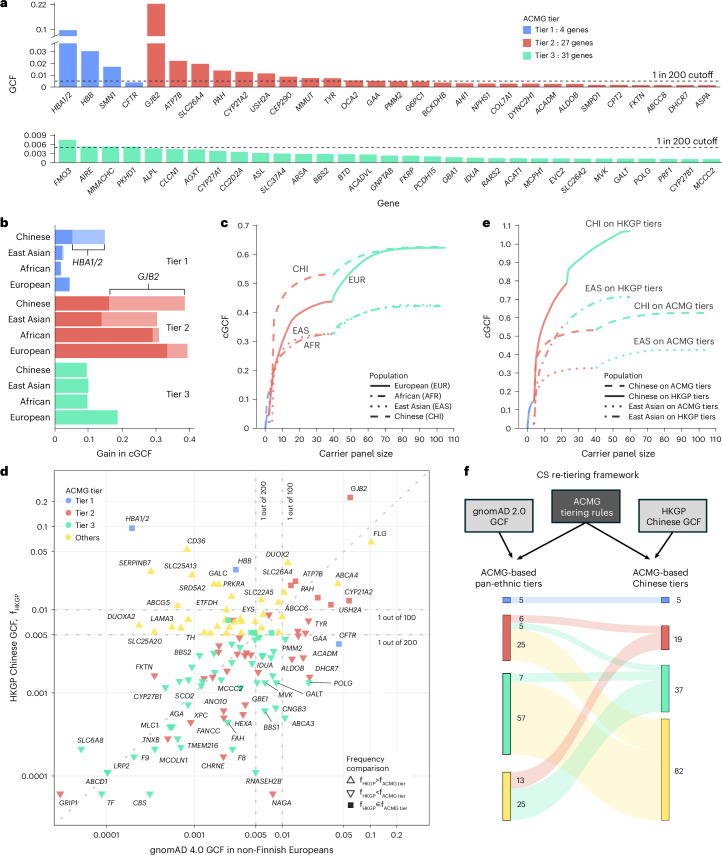
Summary of the findings for recessive genes in the HKGP Chinese cohort (*n* = 18,065). **a**, GCF of ACMG pan-ethnic tier 1−3 CS genes in the HKGP Chinese cohort. **b**,**c**, Contribution of ACMG tier 1–3 genes to the cGCF in the HKGP Chinese cohort compared to other populations from gnomAD 4.0. *HBA1*/*HBA2* and *GJB2*, major contributors to the cGCFs in tier 1 and tier 2, respectively, in Chinese and East Asian populations, are highlighted. The line styles in **c** distinguish the cGCF profiles of different populations. **d**, Comparison of GCF for ACMG and non-ACMG CS genes between HKGP Chinese and non-Finnish Europeans from gnomAD 4.0. **e**, Comparison of cGCF and panel size between ACMG pan-ethnic tiers and Chinese-specific tiers for HKGP Chinese and East Asian populations. **f**, Framework for re-tiering ACMG CS genes for the Chinese population. AFR, African; CHI, Chinese; EAS, East Asian; EUR, non-Finnish European; f_ACMG_ tier, gnomAD 2.0 GCF adopted by the ACMG; f_HKGP_, GCF in the HKGP. Source data

To further investigate whether the ACMG CS gene list is optimal for the Chinese population, we classified the genes in the HKGP Chinese cohort on the basis of its pan-ethnic tiering framework. Only 19 ACMG CS genes exceeded the 1/200 carrier frequency threshold, contributing to a cGCF of 0.62 for 48.1% of carriers (Fig. 3a). *HBA1*/*HBA2* (thalassemia) dominated tier 1 genes. East Asian populations, including HKGP Chinese, showed a relatively high GCF for *GJB2* (Fig. 3b,c); they exhibited lower cGCF for ACMG CS tier 2 and tier 3 genes compared to Europeans when *GJB2* was excluded from the analysis (Supplementary Table 8).

### CS gene re-tiering for Chinese

To increase the CS efficiency in the Chinese population, we included an addition of 1,354 recessive genes from ‘Mackenzie’s Mission’^27^ and other CS panels and re-tiered these CS genes using Chinese-specific GCF data with 1/200 threshold per ACMG framework (designated as HKGP tiers). This resulted in the addition of 38 genes in HKGP tier 2/3, and 82 of the ACMG CS genes were excluded (Supplementary Table 7).

**Extended Data Fig. 3:**
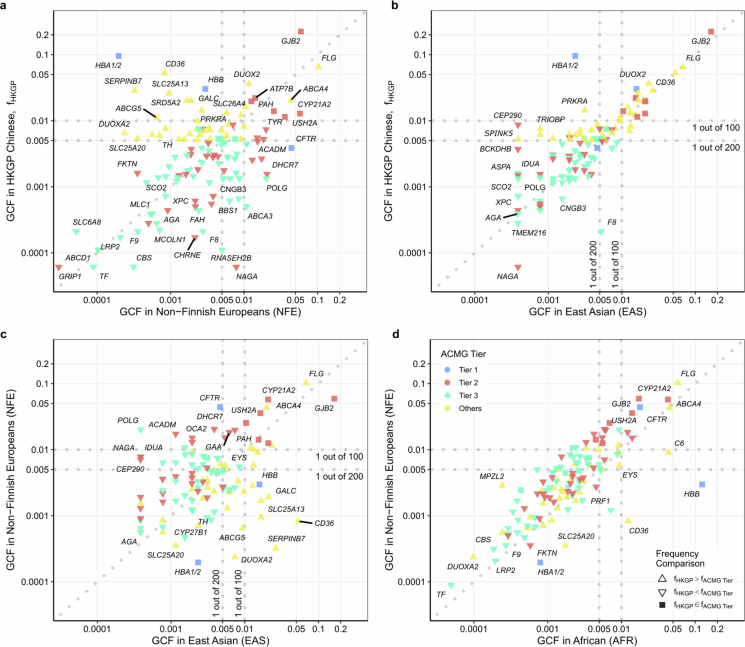
Gene carrier frequency (GCF) comparison across gnomAD 4.0 continental populations and the HKGP Chinese cohort. This figure shows the comparison of GCF for ACMG and non-ACMG carrier screening (CS) genes in the HKGP Chinese cohort versus other continental populations from gnomAD 4.0 (non-Finnish Europeans, East Asians, and Africans). Colours and shapes indicate both the ACMG tier classification of genes and the pairwise GCF comparisons between populations, consistent with Fig. 3d and the ACMG categorization. Subfigures illustrate specific comparisons as follows: **a**, HKGP Chinese vs. non-Finnish Europeans. **b**, HKGP Chinese vs. East Asians. **c**, non-Finnish Europeans vs. East Asians. **d**, non-Finnish Europeans vs. Africans. GCF, gene carrier frequency. Source data

Despite the inclusion of many non-ACMG CS genes with high GCFs in the HKGP Chinese cohort but low GCFs in non-Finnish Europeans (Fig. 3d), the total number of genes decreased from 105 in the ACMG tiers to 61 in the HKGP tiers; the cGCF increased from 0.62 to 1.06, resulting in an increased number of carriers from 8,693 (48.1%) to 11,820 (65.4%) and the cACF from 6.60% to 15.4% (Fig. 3e,f). Similarly, the cGCF for East Asians also increased using HKGP tiers, reflecting a shared genetic background between HKGP participants and East Asian populations (Fig. 3e). By contrast, European profiles showed greater similarity to African^13^ (Supplementary Table 7 and Extended Data Fig. 3). These findings revealed that the pan-ethnic ACMG CS gene list was inadequate for the Chinese population, highlighting an underdetection risk when the unmodified ACMG guidelines were adopted. The HKGP Chinese-specific gene list resolves this gap and indicates broader East Asian relevance through the increased cGCF.

### Functional alleles in pharmacogenomic profiling

**Fig. 4:**
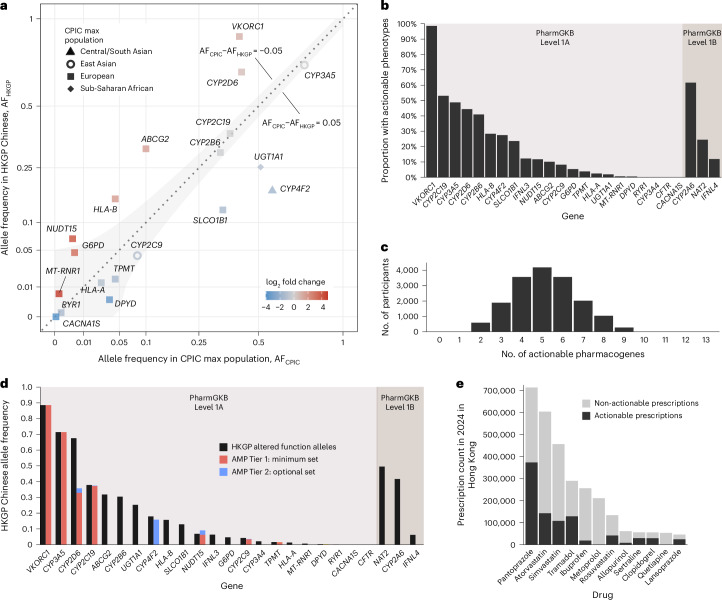
Summary of the findings for pharmacogenes in the HKGP Chinese cohort (*n* = 18,257). **a**, Comparison of altered-function allele frequencies between the HKGP Chinese cohort and the CPIC population with maximum sample size (CPIC maximum population). Altered-function alleles are defined as those with functional differences compared with the CPIC guideline recommendations. Dot shapes denote the CPIC maximum populations; colors indicate fold changes in allele frequencies relative to those of the HKGP. The shaded area, corresponding to the error bands, is defined as the region where allele frequency differences between HKGP and CPIC are less than 0.05. Compared with the CPIC maximum population, the HKGP Chinese cohort presented differences in the frequencies of altered functional alleles across multiple genes. **b**, Proportion of HKGP Chinese individuals with actionable pharmacogenomic phenotypes across pharmacogenes classified as level 1 in PharmGKB’s clinical annotations. A high proportion of HKGP Chinese individuals carried actionable phenotypes for different pharmacogenes, indicating substantial genetic variability with potential clinical impact. **c**, Distribution of the number of actionable pharmacogenes per individual in the HKGP Chinese cohort. Each HKGP participant carried an average of 5.2 actionable pharmacogenomic phenotypes, with individual counts ranging from 0 to 13. **d**, Comparison of altered-function allele frequencies in HKGP Chinese individuals with the AMP-recommended tier 1 and tier 2 allele sets. The AMP tier 1 and tier 2 allele sets comprehensively capture CPIC-defined altered-function alleles in 10 pharmacogenes, whereas coverage remains incomplete for others. **e**, Estimated numbers of actionable and non-actionable prescriptions in Hong Kong in 2024. Predictions were made by multiplying total prescription counts by the frequencies of actionable phenotypes. Actionable phenotypes are predicted to affect nearly 0.9 million prescriptions (30.8% of total) for the 12 most frequently prescribed pharmacogenomic drugs in Hong Kong in 2024. max, maximum. Source data

To analyze the pharmacogenomics—how genetic variation influences drug response—in the Chinese population, we analyzed 25 Clinical Annotation Level 1 A/B pharmacogenes in the Pharmacogenomics Knowledge Base (PharmGKB)^28^, representing highest-evidence tiers for variant−drug associations among all individuals within the HKGP Chinese cohort after excluding four with phenotype-linked bias. We identified 157 altered-function alleles, defined as those with functional differences compared with the recommendations in the CPIC guidelines^17^ across 23 pharmacogenes (Supplementary Table 9). Gene-level comparisons with CPIC population with maximum sample size revealed significant differences in altered-function allele frequencies, with five genes showing GCF > 0.05 and two showing GCF ≤ 0.05 (Fig. 4a). Specifically, the frequency of altered-function alleles of *ABCG2* in our cohort was 31.8%, higher than in other populations, primarily due to an allele with decreased function (rs2231142-T).

Each participant carried an average of 8.78 altered-function alleles. Thirty-nine alleles had a frequency exceeding 0.01, with 17 alleles exceeding 0.10 across 11 pharmacogenes (Supplementary Table 9). Six pharmacogenes (*ABCG2*, *CYP2B6*, *UGT1A1*, *HLA-B*, *SLCO1B1*and *CYP2D6*) exhibited altered-function alleles with frequencies exceeding 0.10 by gene in our cohort, which were not included in the Association for Molecular Pathology (AMP) reportable list^29^. Their frequencies suggest the need for further investigation to determine their potential for reporting under AMP guidelines (for example, CYP2D6*10 + CYP2D6*36 with an allele frequency of 0.36; Fig. 4d).

### Actionable pharmacogenomic phenotypes

Examining metabolic phenotypes from altered-function alleles is a key step toward identifying actionable insights into drug response. Except for *CACNA1S* and *CFTR*, all pharmacogenes had detectable actionable phenotypes, with 14 having actionable phenotype frequencies above 0.10. At least one actionable phenotype was found in 99.98% of the individuals (mean: 5.20 actionable phenotypes per individual; Fig. 4b,c and Supplementary Table 10). These comprise 2.79 ‘therapeutic management’ actionable phenotypes, 1.07 ‘impact-on-safety’ actionable phenotypes and 1.07 ‘impact-on-pharmacokinetic’ actionable phenotypes, categorized by the US Food and Drug Administration (FDA). This high prevalence was largely driven by the variants in *VKORC1*, which affect warfarin sensitivity and the risk of over-anticoagulation and are known to be highly prevalent in Chinese and other Asian populations^7^. *CYP2C19* exhibited high frequencies for two AMP tier 1 no-function alleles (CYP2C19*2: 31.59%; CYP2C19*3: 4.96%), contributing to high actionable phenotypes that require therapeutic changes based on FDA guidelines (Supplementary Table 11).

To assess the potential clinical impact of these findings, we analyzed prescription data for the most prescribed drugs in 2023−2024 from the Hospital Authority, a statutory body that manages all public hospitals in Hong Kong (Supplementary Table 12). Among the top 20 drugs, 12 had guidelines (covering seven pharmacogenes), with pharmacogenomic testing potentially informing nearly one million (903,299/2,936,806, 30.8%) annual prescriptions, mainly for dosage adjustment and alternative therapy (Table 2, Fig. 4e and Supplementary Table 12). Expanding to the top 50 drugs, 13 had FDA-recognized gene−drug interactions, and 16 carried clinically important labels. These findings highlight the opportunity to enhance prescribing practices and improve clinical care, with further research to substantiate their clinical utility.

**Table 2:** Top prescribed drugs with actionable pharmacogenomic phenotypes in Hong Kong (2024)

Rank in 2024	Drug name	Drug type	Prescription count	Actionable phenotype	Major alleles	AMP	Actionable phenotype frequency	Actionable prescription count	Actions (CPIC)		
									Dosage adjustment	Alternative therapy	Clinical monitoring
1	Pantoprazole	Gastrointestinal	713,644	*CYP2C19* UM/IM/PM	IF: *17 NF: *2, *3, *37	Yes	52.30%	373,255	✓		
2	Atorvastatin	Cardiovascular	604,106	*SLCO1B1* DF/PF	NF: *5, *15, *46	No	23.62%	142,682	✓	✓	
3	Simvastatin	Cardiovascular	456,418	*SLCO1B1* DF/PF	NF: *5, *15, *46	No	23.62%	107,800	✓	✓	
6	Tramadol	Anti-inflammatory	289,634	*CYP2D6* UM/IM/PM	IF: *1 × 2, *2 × 2, *1 × 3 DF: *10 + *36, *10, *41 NF: *4, *5, *36	Yes	44.36%	128,472	✓	✓	
7	Ibuprofen	Anti-inflammatory	256,129	*CYP2C9* IM(AS = 1)/PM	DF: *16, *29, *44 NF: *3, *13, *39	Yes	7.20%	18,430	✓	✓	✓
8	Metoprolol	Cardiovascular	210,995	*CYP2D6* PM	NF: *4, *5	Yes	0.16%	347	✓	✓	✓
10	Rosuvastatin	Cardiovascular	133,880	*SLCO1B1* DF/PF	NF: *5, *15, *46	No	31.15%	41,701	✓	✓	
				*ABCG2* PF	rs2231142 (T)	No					
15	Allopurinol	Metabolic	61,063	HLA-B*58:01 carrier	HLA-B*58:01	No	13.43%	8,203		✓	
16	Sertraline	Psychiatric	56,094	*CYP2C19 IM/PM*	NF: *2, *3, *37	Yes	52.30%	29,336	✓	✓	
17	Clopidogrel	Cardiovascular	55,619	*CYP2C19* IM/PM	NF: *2, *3, *37	Yes	52.30%	29,087		✓	
19	Quetiapine	Psychiatric	53,399	*CYP3A4* PM	DF: *18	No	0.03%	18	✓	✓	
20	Lansoprazole	Gastrointestinal	45,825	*CYP2C19* UM/IM/PM	IF: *17 NF: *2, *3, *37	Yes	52.30%	23,968	✓		
	Total		2,936,806					903,299			

AS, activity score; DF, decreased function; IF, increased function; IM, intermediate metabolizer; NF, no function; PF, poor function; PM, poor metabolizer; UM: ultra-rapid metabolizer.

To evaluate potentially deleterious novel pharmacogenetic variants, we analyzed putative protein-disrupting variants in nine pharmacogenes with known loss-of-function (LoF) mechanism. A total of 108 variants were detected in eight genes from 340 (1.86%) individuals. Whereas 88 (81.5%) variants were absent in gnomAD, 81 (75.0%) were unique to single individuals, suggesting a high degree of individual specificity. Notably, 70 (64.8%) variants in *DPYD*, *SLCO1B1* and *G6PD* may harbor a particularly high burden of novel pharmacogenetic variants (Supplementary Table 13). The high prevalence of rare risk and putative protein-disrupting variants in pharmacogenes underscores the need for GS in pharmacogenetic testing, as genotyping may miss or misidentify them.

## Discussion

This study provides a large-scale, integrated genomic analysis specific to the Hong Kong Chinese population^20–22^. Our findings offer guidance for local clinical practice and genetic testing protocols. By establishing a population variant baseline and evaluating clinically actionable genes utility, we fill a major gap in Asian genomic diversity, enabling tailored implementation of diagnostics, screening and pharmacogenomics. Moreover, the comprehensive methodologies and collaborative framework established can serve as a blueprint for other projects to help the development of population-specific genomic resources worldwide.

Previous Chinese precision medicine initiatives, including the Taiwan Precision Medicine Initiative, the China Kadoorie Biobank and pharmacogenomic studies in China, have advanced understanding of common genetic variation and pharmacogenomics, primarily focusing on chronic diseases and drug response using SNP arrays or low-depth GS^30–32^. The HKGP complements these efforts by employing high-depth GS, enabling the study of rare diseases and the identification of novel, complex and structural variants. Together, these initiatives play a vital role in building a comprehensive foundation for precision medicine, with HKGP addressing an important gap by focusing on rare diseases. This flagship study marks a key HKGP milestone, having integrated multidomain genomic analyses through comprehensive GS of more than 20,000 participants.

From our short-read GS biobank and linked phenotypic data, we reveal a 25% diagnostic yield. Consistent with our pilot study and major genome projects^2^, this study demonstrated the scalability of a clinical GS pipeline^18^. Unlike other Asian genome initiatives^8,9^ using targeted approaches, our comprehensive GS with standardized and internationally aligned protocols improves technically challenging variant detection—constituting 15% of P/LP variants in our cohort—that targeted approaches may overlook^33^. To share these findings, including several recurrent founder mutations (Supplementary Table 14), we are establishing gene/variant directories and partnership with the Hospital Authority to integrate HKGP’s GS into clinical genomic testing workflows, mirroring the impactful Genomics England−NHS model. Three years after the pilot, HKGP stands at a critical juncture in genomic findings disclosure. Although our current protocol returns only primary findings^34^, this study serves as the initial step toward broader return options (Supplementary Figs. 1−3).

Beyond its diagnostic applications, we have established a foundational precision medicine resource for the Hong Kong Chinese population. Our local genomic database supports strategic screening programs for dominant genetic disorders and is already being operationalized within the public healthcare system^16^. Although cancer-related mutation burdens have been found to be consistent with other populations, this aggregate masks critical subtype disparities. Lynch syndrome (*MLH1*, *MSH2*, *MSH6* and *PMS2*) demonstrated a substantial local burden, approaching half that of *BRCA1*/*BRCA2*-associated cancers, and is relatively more prevalent in our local population than in Europe (Supplementary Table 6). These findings highlight the underdiagnosis of Lynch syndrome and the need to optimize population-based genetic testing, especially for individuals with a family history^35^. Although Hong Kong has established clear genetic *BRCA1*/*BRCA2* testing criteria (https://www.chp.gov.hk/files/pdf/breast_cancer_professional_hp.pdf), Lynch syndrome screening remains underdeveloped, warranting strategic review and implementation to improve cancer prevention.

**Extended Data Fig. 4:**
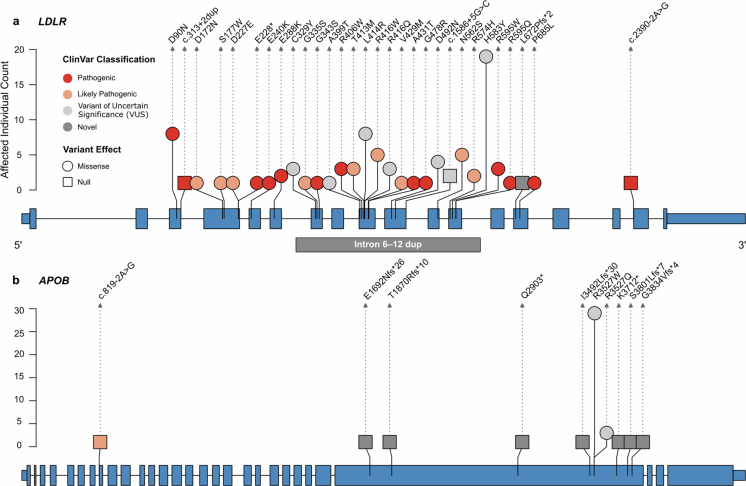
Pathogenic or likely pathogenic mutation spectra for *LDLR* and *APOB*. **a**, P/LP variants identified in *LDLR* from the HKGP Chinese cohort. **b**, P/LP variants identified in *APOB* from the HKGP Chinese cohort. Exons are depicted as blue boxes linked by thin lines (introns); the grey bar under *LDLR* indicates a single intron 6–12 duplication event that we observed. Circles denote missense variants, and squares denote null variants. The colour of a symbol corresponds to the ClinVar classification (red = pathogenic; orange = likely pathogenic; light grey = VUS; dark grey = novel). P/LP: pathogenic or likely pathogenic. Source data

Unlike other populations, nearly half of our individuals at risk of dominant disorders were from cardiovascular function-associated genes—that is, cardiomyopathy (*TTN* and *DSG2*), arrhythmia (*SCN5A*) and hyperlipidemia (*LDLR* and *APOB*). This, alongside the absence of P/LP variants in over one-quarter of the ACMG secondary finding genes, necessitates prioritized cardiovascular screening and resource allocation. Given that heart diseases were the third leading cause of deaths in Hong Kong (https://www.chp.gov.hk/en/healthtopics/content/25/57.html), our findings urge policy shifts, including adult cardiology genetic testing for sudden death risks (*SCN5A* and *DSG2*), pediatric cardiology expansion beyond congenital disorders and population screening for hyperlipidemia genes (*LDLR* and *APOB*) (Extended Data Fig. 4). As for familial hypercholesterolemia, current local genetic testing includes *PCSK9*, *LDLR* and *APOB*^36^, whereas our data revealed minimal *PCSK9* variants in our Chinese population. To optimize resource utilization, we recommend refocusing genetic testing on high-yield genes (*LDLR* and *APOB*) to improve familial hypercholesterolemia management efficiency. Our findings support policy development to refine cardiac genetic services and implement screening pilots for high-burden conditions, advancing HKGP’s objectives of enhancing personalized disease risk prediction with Chinese-specific genomic resources.

Nearly half of HKGP participants carried P/LP variants from the ACMG pan-ethnic CS gene list, translating to an estimate of one in 16 couples at risk of having offspring affected by recessive disorders. Consistent with previous studies^37,38^, the risk coverage for ACMG CS genes was significantly lower in the HKGP Chinese cohort than in the European cohort (Supplementary Table 12). Clinically significant recessive conditions with high ACFs, such as *CD36*-associated bleeding disorders and *FLG*-linked ichthyosis vulgaris, were not properly covered. To address these patterns, we re-tiered CS genes using the ACMG guidelines weighted by HKGP Chinese carrier frequencies. This doubled the risk coverage from 6.6% to 15.4%, translating to 3,229 at-risk pregnancies annually in Hong Kong, with 42% fewer genes screened. Specifically, 14 of the 38 additionally included genes are associated with metabolic disorders. Although the newborn screening program in Hong Kong typically prioritizes such conditions, it includes only seven genes (Supplementary Table 7). Expanding coverage to include all 14 genes would enable early intervention potentially for approximately 130 at-risk pregnancies annually. Other than metabolic disorders, some conditions were overlooked with the current screening panels. Omissions of the related genes can lead to potentially devastating consequences, including immediate mortality risks (*GALC*, *F7*and *C6*), irreversible disability (*CAPN3*and *TH*), chronic debilitation (*DNAH11*, *LAMA3*and *SPINK5*) and quality-of-life impacts (*PRKRA*and *EDA*). In addition, we further identified a residual ‘long tail’ risk from genes not covered in Chinese CS tiers 1−3 and undetectable by conventional panels alone. These findings from re-tiering support updating the population-specific CS gene list to improve efficiency with fewer resources, underscoring the role of HKGP in guiding population-optimized reproductive genomic policy.

In characterizing pharmacogenomic alleles, GS demonstrated superior performance, especially for genes such as *CYP2D6*, where CNVs and SVs significantly impact function. This enhanced resolution revealed frequency disparities in clinically consequential pharmacogenetic alleles. The HKGP dataset provided validation for the AMP recommendations, exemplified by *CYP2C19*, where AMP tier 1 alleles accounted for 98.41% of functional alterations, whereas tier 2 burden (0.57%) was largely driven by the Chinese-specific CYP2C19*37 allele (0.38%). Although relatively rare, its potential clinical relevance is amplified by the high volume of prescriptions for drugs metabolized by *CYP2C19*, including sertraline, clopidogrel and lansoprazole.

Locally prevalent variants, shaped by population-specific prescribing patterns, can prevent adverse drug reactions and improve therapeutic outcomes for patients, when incorporated into local guidelines. Our HKGP resource addresses potential clinical population-specific needs, as we revealed that 30.8% (0.9 million) of common annual prescriptions involve drugs with pharmacogenetically actionable phenotypes (Supplementary Table 12). Only three of the 20 most prescribed drugs carry FDA-recognized gene−drug interactions with therapeutic management recommendations, and 16 of the top 50 drugs carry FDA clinically important labels; this regulatory threshold represents a floor, not a ceiling, for clinically actionable pharmacogenomics.

Although this study provides foundational genomic insights for Hong Kong, its generalizability may be limited by the small sample size. To ensure the robustness of future research findings, we will expand our biobank diversity to enhance population representativeness. The present study relies on short-read sequencing, which has recognized limitations in detecting SVs and resolving repetitive genomic regions. Building upon our existing workflows and internationally standardized variant interpretation^19^, we plan to integrate advanced technologies such as long-read sequencing and multiomics approaches^39,40^ to overcome these challenges, alongside continued advances in bioinformatics^18^, and to improve efficiency and diagnostic power^41^. For translation of clinical actionability, HKGI will continue collaborating with the Hospital Authority and international research initiatives to develop interoperable clinical decision supports and pragmatic trials to validate their utility in primary care. For pharmacogenomics, additional work, such as the PREPARE clinical trial^42^, is needed to link population-specific variants to clinically meaningful drug response or adverse effect profiles (for example, effect sizes, penetrance and outcomes in real-world care) before they can be considered for guideline implementation. Moreover, newborn screening studies will be implemented to validate risk estimates for recessive disorders. These efforts establish the project as a key resource for precision medicine.

The HKGP represents a paradigm shift from reactive symptom management to proactive health preservation by delivering precision-guided clinical applications, including optimized screening, prevention, therapeutic strategies and reproductive pathways. This study provides a large-scale genomic analysis tailored to the underrepresented Chinese population of Hong Kong, building the scientific foundation to redesign clinical services around predictive risk profiling. These insights drive lifecourse-optimized personalized care, cross-generational planning and systemic healthcare evolution through evidence-based policy, positioning Hong Kong at the vanguard of precision medicine—where genomics underpins clinical decision-making, public health strategy and societal wellbeing. As we expand our efforts, this study serves as both a foundation and a bridge to future genomic medicine advancements in Hong Kong and beyond.

## Methods

Ethical approval for the HKGP and this study was granted by the central institutional review board (IRB) (HKGP-2021-001 and HKGP-2022-001) and the IRBs of the Department of Health (L/M257/2021), the Joint Chinese University of Hong Kong/New Territories East Cluster (2021.423 and 2023.120) and the University of Hong Kong/Hospital Authority Hong Kong West Cluster (UW 21–413 and UW 23–289).

### Participants

For the HKGP, both asymptomatic individuals and symptomatic probands suspected of having a genetic disease were prospectively identified and recruited across a range of medical specialities at the three partnering centers of the HKGI. All participants received pretest genetic counseling and provided informed written consent following the unique three-tier consent and assent model designed by the HKGI^43^. As described in our pilot study, detailed phenotype information, including family history and symptom onset, was collected and recorded using HPO terms^18^.

HKGP participants whose samples were subjected to GS, variant calling and classification before November 2024 were included in this study^18^. Probands with suspected genetic disease(s), together with their family members, and who had finished genetic diagnosis were included in the diagnostic cohort. Unrelated Chinese participants, including both healthy and affected singletons, in addition to parents from duo and trio family structures, were included for the analysis as the HKGP Chinese cohort. Notably, individuals exhibiting phenotypes associated with selected dominant genes (described in the Gene selection section below), as well as participants with offspring demonstrating phenotypes related to selected recessive genes, were excluded from the respective analyses. To ensure unrelatedness, PLINK (version 2.0) was employed to assess the biological sex and the relatedness among the remaining participants in the HKGP Chinese cohort, with one participant removed from each pair (parents will be retained for non-singleton participants) exhibiting a kinship coefficient greater than 0.177 (ref. ^41^). Participants with conflicting self-reported sex and sequencing data imputed sex (PLINK 2 --impute-sex) were removed for this study. Chinese ethnicity was determined on the basis of self-reported data and validated through ancestry admixture analysis, where Chinese ethnicity was identified as the predominant ancestry using SNVstory^44^.

### Enrollment criteria

1. Undiagnosed disorders
  - The definition for undiagnosed disorders is disorders without a specific diagnosis after thorough evaluation through clinical assessment and routine investigation.
  - HKGP will recruit patients who meet the following criteria:
    i) The patient has a medical condition that meets the aforesaid definition.
    ii) Consent of the patient is obtained for providing and sharing medical information and samples.
    iii) The patient (or parents or legal guardian) agrees to trio testing—that is, blood sample to be taken from patient and both parents. In case trio testing is not possible, the decision will be made based on the relevant specialists’ assessment.
2. Cancers with clinical clues linked to possible hereditary components
  - The definition is as follows:
    i) Having more than one first-degree or second-degree relative with confirmed cancer; or
    ii) Developing cancer at a younger age than expected for that cancer type; or
    iii) Pediatric patients with cancer; or
    iv) Having more than one type of cancer in the same person
  - Recruitment criteria for patients with hereditary cancer and genetic predisposition to cancer would be:
    i) The patient is pathologically confirmed with cancer that meets the above definition; and
    ii) Consent of the patient is obtained for providing and sharing medical information and samples.
3. Other patients who will benefit from GS (under the theme ‘Genomics and Precision Health’ of the main phase of HKGP)
  - ‘Genomics and Precision Health’ is a cohort that aims to improve the health of individuals with and without specific diseases by harnessing the power of genomics technologies. The health of individuals can be improved by genomics technologies according to clinical, personal, economic and system utilities.
4. Unaffected first-degree family members aged older than 18 years of the above three cohorts

### Exclusion criteria

Exclusion criteria include patients with known genetic cause for their condition or patient/parents/legal guardian/substitute decision-maker unwilling to participate in the study.

### GS and variant detection

The detailed workflows for sequencing and data analysis of short-read GS were previously described^18^. In brief, whole blood (or buccal/saliva when necessary) was collected, and genomic DNA was extracted for polymerase chain reaction (PCR)-free short-read GS using the KAPA HyperPlus Kit and sequenced on Illumina NovaSeq 6000 or X Plus to achieve a mean coverage of ≥29.5×. After passing quality control checks, the GATK-based standard bioinformatics pipeline was used for secondary analysis. In short, reads were aligned to the GRCh38 reference using BWA (version 0.7.17) with duplicate removal via Picard (version 2.27.4), and variant calling for autosomes, sex chromosomes and the mitochondrial genome was performed using GATK HaplotypeCaller, Mutect2 (version 4.2.6.1), CNVKit (version 0.9.9), Manta (version 1.6.0) and ExpansionHunter (version 3.1.2) to detect SNVs, indels, CNVs, SVs and STRs^45–48^.

### Gene selection

Genes with strong or definitive gene‒disease associations, as classified by Clinical Genome Resource (ClinGen) (‘definitive’ or ‘strong’), Genomics England PanelApp or PanelApp Australia (‘green’), were prioritized. Genes with moderate evidence of association (‘moderate’ in ClinGen or ‘amber’ in PanelApp) were selectively included on the basis of consensus with referring clinicians.

For the dominant disorder-related genes used for the HKGP Chinese cohort analysis, we adopted a reference gene list of 73 dominant genes from the ACMG secondary findings gene list version 3.2 (ref. ^16^).

For recessive disorder-related genes, we consolidated a comprehensive list of 1,459 genes from multiple well-recognized sources to ensure broad coverage and clinical relevance. These sources included (1) 105 genes from the ACMG-recommended CS pan-ethnic gene list, including *HBA1* and *HBA2* for Asian individuals^15^; (2) 1,283 genes from ‘Mackenzie’s Mission’ version 2.2 gene list, derived from a large-scale Australian CS initiative^27^; (3) 101 autosomal recessive genes associated with treatable inherited disorders^49^; and (4) 140 additional genes from other commercially available CS panels and relevant published resources. This integrative approach was intended to maximize the clinical utility of our CS protocol by capturing both established and emerging gene‒disease associations. Detailed lists of the dominant and recessive genes are provided in Supplementary Tables 6 and 7.

### Variant classification

#### SNVs and indels

##### Diagnostic cohort

Following a phenotype-driven diagnostic workflow similar to that used in the HKGP pilot study^18^, SNVs and indels (<50 base pairs) with allele frequencies <0.005 in gnomAD versions 2.1.1 and 3.1.2 were prioritized via inheritance-based filtering and phenotypic matching with HPO terms through Exomiser^50^, supplemented by virtual gene panels from Genomics England PanelApp and PanelApp Australia as described above. The pathogenicity of the variants was determined according to ACMG guidelines and up-to-date recommendations from the ClinGen Sequence Variant Interpretation (SVI) Working Group through manual curation. Specifically, mitochondrial variants were analyzed according to the ClinGen Mitochondrial Disease Nuclear and Mitochondrial Expert Panel Specifications to the ACMG/AMP Variant Interpretation Guidelines. Following the HKGP principles of reporting, we reported variants that were classified as P/LP only when their biological effects matched the patient phenotype. Orthogonal validation was performed for all P/LP variants using independent DNA extracted from the original sample. Variants of uncertain significance (VUSs) in dominant genes that meet the following criteria, agreed upon by all parties in the multidisciplinary team, including clinicians, were reported: highly compatible with the clinical phenotypes and when additional secondary assay/analysis—such as RNA sequencing, enzyme activity testing, immunohistochemical staining, imaging studies and segregation analysis—can be performed to confirm the diagnosis. Variants were visualized using Integrated Genomics Viewer (IGV) version 2.17.4 (ref. ^51^).

##### The HKGP Chinese cohort (recessive and dominant genes)

In addition to diagnostic findings, SNVs and indels in our consolidated gene lists for other clinical findings were retained for curation if their allele frequencies were <0.05 in gnomAD version 3.1.2 unless they were included on the BA1 (‘standalone benign’) criterion exception list. Through a combination of automated and manual curation (Supplementary Fig. 4), these variants were classified into three categories: reported P/LP, ACMG P/LP and ACMG VUS or benign (ACMG VUS-B).

- Reported P/LP
  P/LP variants from ClinVar with three-star or four-star review status were classified by expert panels such as ClinGen or authoritative consortia such as the Evidence-based Network for the Interpretation of Germline Mutant Alleles (ENIGMA). In addition, to reduce the total number of variants for manual review, one-star or two-star review status variants were also classified as reported P/LP for recessive genes.
- ACMG P/LP and VUS or benign (VUS-B)
  Other identified variants were processed through two analytic pipelines: (1) both ClinVar-reported and novel variants in the dominant gene list were classified using ACMG/ClinGen guidelines and a Bayesian classification framework; (2) ClinVar-unreported null variants in the LoF genes were classified using the PVS1 criterion. All ClinVar data were accessed and extracted on 30 June 2024.

For the variants detected in the HKGP Chinese cohort, the classification process was further refined using our previously established semiautomated brief cohort analysis workflow (S-BCAW)^52^. Both automated scoring and manual curation were applied throughout the curation process. For recessive genes, null variants absent from ClinVar were assigned PVS1 criterion using AutoPVS1 (version 1.1) and classified similarly^21^.

#### SVs and CNVs

##### Diagnostic cohort

A phenotype-driven diagnostic workflow similar to that used in the HKGP pilot study was followed. The pathogenicity of deletions and duplications was interpreted in accordance with the joint consensus standards of CNV interpretation by the ACMG and ClinGen^53^. Currently, there is no established expert consensus for the interpretation of other SV types. For these variant types, PVS1 was applied at an appropriate strength on the basis of the predicted impact on gene function^54^.

##### The HKGP Chinese cohort (recessive and dominant genes)

The analysis of SVs focused specifically on genes identified in the predefined gene list, where the disease mechanism is LoF. Insertions, deletions and duplications within these gene lists were curated according to the ACMG/ClinGen joint consensus guidelines for CNV interpretation^53^.

Among the recessive disorder-related genes, some loci present unique technical challenges that cannot be reliably detected by conventional variant callers, as described above. To overcome these limitations, specialized approaches were employed: an in-house developed caller was used for detecting common α-globin gene deletions (*HBA1*/*HBA2*), and Illumina’s SMNCopyNumberCaller was used for precise quantification of *SMN1* and *SMN2*(ref.^55^).

### STRs

STRs were analyzed at loci defined by the Illumina repeat catalog (https://github.com/Illumina/RepeatCatalogs). STR calls were considered pathogenic if the repeat size was greater than the pathogenic reportable threshold summarized in gnomAD on the basis of the literature.

### Defining GCF and cGCF

To characterize carrier frequencies at the gene level, we adopted the concept of GCF, defined as the fraction of participants carrying any P/LP variant(s) in the gene.

To facilitate further analysis across groups of genes, we introduced the concept of cGCF, which is defined as the sum of GCFs for all genes within a specific gene list or tier. These metrics provide a robust framework for quantifying carrier frequencies at multiple levels of granularity, enabling population-specific insights and facilitating tier-based gene classification.

### Clinical utility

Clinical utility is defined as the percentage of individuals experiencing potential changes to clinical management after a diagnosis, which helps to accelerate decision-making and the consensus formulation process for all relevant stakeholders. The potential change in clinical management was classified into seven categories according to Riggs et al. and the UK 100,000 Genomes Project^19,56^: (1) referral to specialist(s); (2) indication for further diagnostic tests to evaluate possible complications; (3) initiation or contraindication of interventional or surgical procedures; (4) surveillance for potential future complications; (5) initiation or contraindication of medications; (6) lifestyle changes; and (7) clinical trial eligibility (meet enrollment criteria for phase 2 or higher interventional (related to drugs, medical devices, procedures and vaccines as defined in https://clinicaltrials.gov/) or observational (focused on assessing non-interventional biomedical or health outcomes) trial studies listed in https://clinicaltrials.gov/ or https://www.clinicaltrialsregister.eu/ that were related to the patient’s target gene and disease at the time of diagnosis).

### Diagnostic odyssey

The diagnostic odyssey is defined as the time from when the disease’s symptoms are first noted in the proband (odyssey start date) to the time when a genetic diagnosis is reached. We determined the odyssey start date by retrieving the earliest record in the clinical management system that describes the symptoms of the primary indication(s) when referred to the HKGP. The date of genetic diagnosis was determined on the basis of the date at which the HKGP issued the report to the referring clinician. The diagnostic odyssey was calculated as the date of genetic diagnosis minus the odyssey start date, rounded to the nearest year; for odysseys shorter than 1 year, duration was calculated in months.

### Founder mutation screening

Novel potential founder mutations were assessed in this study. The following selection criteria were applied for novel founder mutations: (1) repeated occurrence among the participants in this study, (2) absence in the gnomAD non-East Asian genome dataset and (3) absence in ClinVar. For known variants, Chinese-specific founder mutations were directly collected from the literature and compared with our findings. Shared haplotype analysis was conducted for both novel and known potential founder mutation loci among related participants carrying the mutation. This analysis used IBDseq^57^ for common variants (minor allele frequency >0.5% in this study).

### Estimation of ACF

To estimate the ACF, all possible mating combinations among unrelated Chinese participants included in this study were evaluated. Specifically, (1) all pairings, irrespective of sex, were considered for autosomal recessive genes (${C}_{2}^{n}$ pairings in total; *n* is the number of unrelated Chinese participants), and (2) only female‒male pairings were assessed for X-linked genes. A virtual couple was classified as ‘at-risk’ if both individuals carried P/LP variants in any of the same autosomal recessive genes or if the female carried P/LP variants in any X-linked genes. The ACF estimated through random mating was then compared to the observed frequency of actual couples carrying P/LP variants in the same gene within this cohort.

### Re-tiering CS genes based on ACMG guidelines for the Chinese population

Genes were re-tiered on the basis of ACMG CS guidelines, with carrier frequency thresholds applied to the gene-specific GCF derived from Chinese population data in the HKGP. Tier 1 was unchanged and includes *CFTR*, *SMN1*/*SMN2*, *HBA1*/*HBA2* and *HBB*. Tier 2 included genes associated with severe or moderate phenotypes and a carrier frequency of at least 1/100 in autosomes in our Chinese population, whereas tier 3 included genes with carrier frequencies of at least 1/200 in sex chromosomes or autosomes. This tiering approach was designed to reflect population-specific genetic characteristics while maintaining consistency with the ACMG’s evidence-based recommendations. cGCFs for different tiers were compared for this Chinese tier and ACMG pan-ethnic tiers for the Chinese population and other populations in the gnomAD 4.0 database^13^.

### Pharmacogenomics

#### Gene selection and individual selection

To profile the actionable pharmacogenomic variants, we consolidated a gene list of 25 pharmacogenes with PharmGKB Clinical Annotation Level 1A or 1B (Supplementary Table 9). Among the 25 pharmacogenes analyzed, seven pharmacogenes (*CACNA1S*, *CFTR*, *DPYD*, *G6PD*, *MT-RNR1*, *RYR1*and *VKORC1*) are associated with congenital diseases as classified by ClinGen with definitive, strong or moderate gene−disease validity or as ‘green’ (diagnostic) or ‘amber’ (borderline) in relevant disease panels in Genomics England PanelApp and PanelApp Australia (similar gene selection approach for the diagnostic cohort). To avoid confounding effects from these conditions, individuals from the HKGP Chinese cohort were excluded from the analysis if their own or their offsprings’ primary phenotypes matched the associated congenital diseases. The remaining individuals were included for the pharmacogenomic analysis of known alleles and novel variants.

#### Known pharmacogenomic variants

Genotyping of known alleles of the 25 selected pharmacogenes was conducted using various tools: (1) Cyrius version 1.1.1 (ref. ^58^) for *CYP2D6* alleles, (2) HLA-HD version 1.7.0 (ref. ^59^) for *HLA-A* and *HLA-B* alleles, (3) Aldy version 4.6 (ref. ^60^) for other pharmacogenes with star allele nomenclature and (4) VCF-derived for pharmacogenes defined by dbSNP rsIDs. Allele function and phenotype were determined on the basis of information sourced from CPIC and PharmGKB (accessed 12 November 2024). Variants listed in the AMP’s minimum sets for pharmacogenomic testing are also labeled in the same table.

To investigate the discrepancy between the Chinese population and the population with maximum sample size in CPIC, we followed the definitions and methods described by Hernandez et al.^17^ to compare the differences in the frequencies of altered functional alleles.

To further investigate the significance of the clinical impact of the actionable phenotypes in pharmacogenes, we categorized actionable phenotypes according to the three sections defined by the FDA Tables of Pharmacogenetic Associations (www.fda.gov/medical-devices/precision-medicine/table-pharmacogenetic-associations) (Supplementary Table 11).

#### Novel variants in LoF pharmacogenes

To further investigate novel deleterious variants in pharmacogenes, SNVs, indels, CNVs and SVs were detected using the same methodology described earlier. This analysis focused on nine pharmacogenes for which no-function alleles have been defined to be associated with actionable phenotype by CPIC or PharmGKB (*CYP2B6*, *CYP2C9*, *CYP2C19*, *CYP2D6*, *DPYD*, *G6PD*, *NUDT15*, *SLCO1B1*and *TPMT*). These genes were selected based on the rationale that LoF is a mechanism associated with their actionable phenotype. Only putative protein-disrupting variants, including frameshift, inframe, splicing and nonsense variants in these genes with PVS1 strength reaching ‘very strong’ from AutoPVS1, were included in this study after manual investigation on IGV for to ensure high-quality variants.

#### Estimated actionable prescriptions in Hong Kong

To examine the pharmaceutical landscape in Hong Kong, the prescription records of all medications from hospitals under the Hong Kong Hospital Authority between 1 December 2023 and 30 November 2024 were retrieved from the Clinical Data Analysis and Reporting System (CDARS) database. The top 50 drugs were selected on the basis of the total prescription count during this period. We estimated the number of actionable prescriptions by multiplying the frequency of pharmacogenomic actionable phenotypes, as defined in PharmGKB and CPIC and identified in HKGP’s data, for each individual pharmacogenomic gene. To further study the clinical relevance, we analyzed these prescribed drugs using the FDA’s Table of Pharmacogenomic Biomarkers in Drug Labeling (www.fda.gov/drugs/science-and-research-drugs/table-pharmacogenomic-biomarkers-drug-labeling) and identified clinically consequential pharmacogenomic information with three key labeling sections: adverse reactions, warnings and precautions and dosage and administration.

### Results reporting

#### Primary findings

Building upon patient and clinician feedback, we will continue to prioritize returning clinically significant findings directly related to the referral indication and clinical phenotype.

#### Additional medically actionable findings

##### Dominant disorders

For participant opt-in for feedback of additional findings of GS, we developed a plan for reporting and returning findings in 13 genes (of which 12 are associated with dominant disorders)—*MLH1*, *MSH2*, *MSH6*, *MUTYH*, *APC*, *BRCA1*, *BRCA2*, *VHL*, *MEN1*, *RET*, *LDLR*, *APOB*and *PCSK9*—based on clinical actionability and severity. In compliance with ACMG guidelines and reporting guidance, only P/LP variants will be reported (https://search.clinicalgenome.org/kb/genes/acmgsf). This structured approach ensures responsible return of high-impact genetic information while respecting clinical context and participant preferences.

##### Recessive disorders

For reporting and returning additional findings of MUTYH-associated polyposis, only individuals with two identified disease-causing variants will receive results. Regarding expanded CS, we are at the crossroads. Although we will continue to return carrier status upon patient request, this study reinforces our decision to develop a Chinese-specific CS panel rather than relying solely on resources based on European ancestries, such as ACMG and ‘Mackenzie’s Mission’. We have demonstrated our capability to identify and return these results to patients.

##### Pharmacogenomics

Given the potential for broad impact, we are now initiating comprehensive review with our scientific and ethics advisory committees to explore strategies for pharmacogenomics implementation.

### Statistics and reproducibility

All statistical analyses were performed using R version 4.3.3. Diagnostic yield comparisons for the diagnostic cohort and cGCF comparison in recessive genes were performed by the one-sided *χ*^2^ test (Extended Data Table 2 and Supplementary Table 8).

ACF comparisons were performed by two-sided Fisher’s exact test for each gene, and the *P* value was further corrected by Bonferroni correction for multiple testing on multiple genes (Supplementary Table 7). The significance level was set as *P* < 0.05 for all analyses in this study.

No statistical method was used to predetermine sample size. The sample size for the diagnostic cohort was determined by including all the HKGP participants who finished genetic diagnosis by November 2024 in HKGI. The sample size for the HKGP Chinese cohort was determined by including all unrelated Chinese participants who finished variant analysis by the same cutoff date.

For both cohorts, individuals with sequencing data who failed the quality control were excluded in this study. The experiments were not randomized. The investigators were not blinded to allocation during experiments and outcome assessment.

### Reporting summary

Further information on research design is available in the Nature Portfolio Reporting Summary linked to this article.

## Online content

Any methods, additional references, Nature Portfolio reporting summaries, source data, extended data, supplementary information, acknowledgements, peer review information; details of author contributions and competing interests; and statements of data and code availability are available at 10.1038/s41591-026-04410-w.

## Supplementary information

Supplementary Information Supplementary Figs. 1−4 and legends for Supplementary Tables 1−14.

Reporting Summary

Peer Review File

Supplementary Tables Supplementary Table 1: Full list of probands in the diagnostic cohort, including clinical information and genetic diagnoses. Supplementary Table 2: Variant details and clinical management of positively diagnosed probands included in the diagnostic cohort. Supplementary Table 3: Identified P/LP SNVs and indels of dominant and recessive genes in the HKGP Chinese cohort. Supplementary Table 4: Identified P/LP SVs, CNVs and STRs in dominant and recessive genes in the HKGP Chinese cohort. Supplementary Table 5: Reclassified P/LP variants in the HKGP Chinese cohort. Supplementary Table 6: GCF of P/LP variants in dominant disorder-related genes. Supplementary Table 7: GCF of P/LP variants and CS tier in recessive disorder-related genes. Supplementary Table 8: Statistical comparison between two cGCFs from different populations and tiering sources. Supplementary Table 9: Frequencies of altered-function alleles for pharmacogenes. Supplementary Table 10: Number of actionable metabolomic phenotypes for pharmacogenes per participant (source data for Fig. 4c). Supplementary Table 11: Frequency of metabolomic phenotypes for pharmacogenes. Supplementary Table 12: Top 50 most prescribed drugs in Hong Kong with FDA drug labels and pharmacogenetic associations. Supplementary Table 13: Novel putative protein-disrupting variants in LoF pharmacogenes. Supplementary Table 14: Novel founder mutations found in the HKGP with shared haplotypes.

## Source data

Source Data Figs. 1−4 and Extended Data Figs. 1−4 Statistical Source Data for Figs. 1–4 and Extended Data Figs. 1−4.

## Figures and Tables

**Extended Data Table 1:**
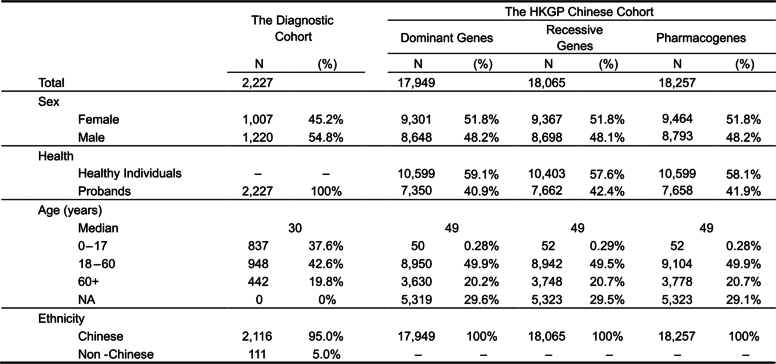
Participant demographics

**Extended Data Table 2:**
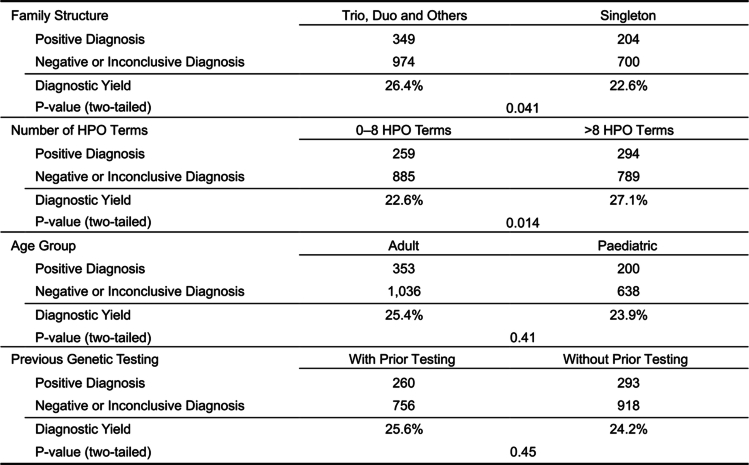
Determinants of diagnostic yield in the diagnostic cohort

**Extended Data Table 3:**
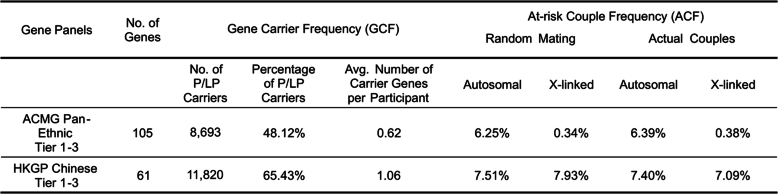
Cumulative gene carrier frequency and at-risk couple frequency in the recessive disorder-related gene panels

A couple is considered at-risk if they both have P/LP variants in the same recessive disorder genes, or if mother has P/LP variant in X-linked recessive disorder genes. P/LP, pathogenic or likely pathogenic.

## Data Availability

Deidentified proband-level information used for the diagnostic cohort is available in Supplementary Tables 1 and 2. Detailed variant-level information used for the diagnostic cohort and the HKGP Chinese cohort is available in Supplementary Tables 3, 4, 5, 9, 13 and 14. Detailed gene-level information is available in Supplementary Tables 6, 7 and 11. Variants identified in the diagnostic cohort were uploaded to ClinVar in batches (https://www.ncbi.nlm.nih.gov/clinvar/submitters/510250/). Deidentified individual-level genotype data of variants presented in this paper and additional aggregate-level data not included in this paper are currently available to researchers who obtain IRB approval by completing the following steps: 1. Researchers should submit a Data Access Request to HKGI (hkgi_gc_team@genomics.org.hk) outlining the proposed research, including its purpose, scope of data to be accessed and researcher information. 2. The HKGI Data Access Review Panel will review the application in a quarterly meeting to assess the scientific, clinical, technical, resource and regulatory feasibility of the proposal. All feasible proposals will be approved. 3. The HKGI team will collaborate with applicants to prepare the formal proposal and related IRB documentation. 4. Anonymous, aggregate data will then be provided to applicants either directly or within designated HKGI facilities (for 3−12 months), depending on the assessment of the proposal. The same application process also applies to other individual-level genomic data beyond this paper. As the HKGP is actively recruiting new participants at the time of writing, access to such data will be granted to external researchers after the completion of the main phase of this project in 2030. Source data are provided with this paper.

## Code availability

The code and scripts used to perform all analyses and generate the figures in this study are publicly available on GitHub at https://github.com/hkgi-steam/hkgi_flagship_paper_2025. The repository includes analysis scripts for identifying variants, generating summary statistics and producing the display figures. Instructions for reproducing the figures are also provided, including steps to build the required computational environment using Jupyter Notebook and Apptainer.
