# Supplementary material for: Population-scale genomic medicine with the Hong Kong Genome Project
Source: Nat Med. 2026 May 15;32(6):2277–87. doi: 10.1038/s41591-026-04410-w (PMC13278956; doi:10.1038/s41591-026-04410-w)
Supplement: Supplementary file 1 — Supplementary Figs. 1−4 and legends for Supplementary Tables 1−14. [file 41591_2026_4410_MOESM1_ESM.pdf]

nature medicine

Supplementary information

https://doi.org/10.1038/s41591-026-04410-w

Population-scale genomic medicine with the Hong Kong Genome Project

In the format provided by the authors and unedited

Supplementary Information

Supplementary Figures

Supplementary Figure 1: Joint clinical findings for HKGP003648-1

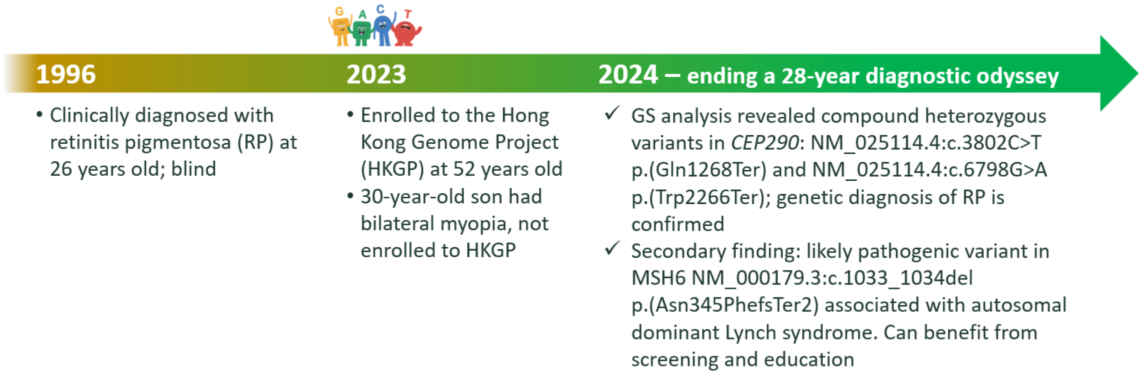

Clinical and genetic timeline of a 52-year-old female diagnosed with retinitis pigmentosa (RP) at age 26 and referred for evaluation after becoming completely blind. Singleton genome sequencing (GS) identified compound heterozygous pathogenic nonsense variants in the *CEP290* gene (c.3802C>T p.(Gln1268Ter) and c.6798G>A p.(Trp2266Ter)), confirming a diagnosis of autosomal recessive *CEP290*-related ciliopathy, a condition with phenotypes ranging from isolated retinal dystrophies to severe syndromic ciliopathies. Additionally, GS analysis revealed a likely pathogenic variant in the *MSH6* gene (c.1033_1034del p.(Asn345PhefsTer2)), associated with autosomal dominant Lynch syndrome, a hereditary cancer predisposition syndrome. Although the proband exhibited no Lynch syndrome phenotypes, preventive screening for colorectal cancer and education on the risk of endometrial cancer (EC) were recommended, with the option of prophylactic hysterectomy to mitigate EC risk. Cascade testing for the *MSH6* variant was advised for the proband's 30-year-old son, who may consider colonoscopy screening starting at age 35 if found to carry the variant.

Supplementary Figure 2: Joint clinical findings for HKGP001994-1

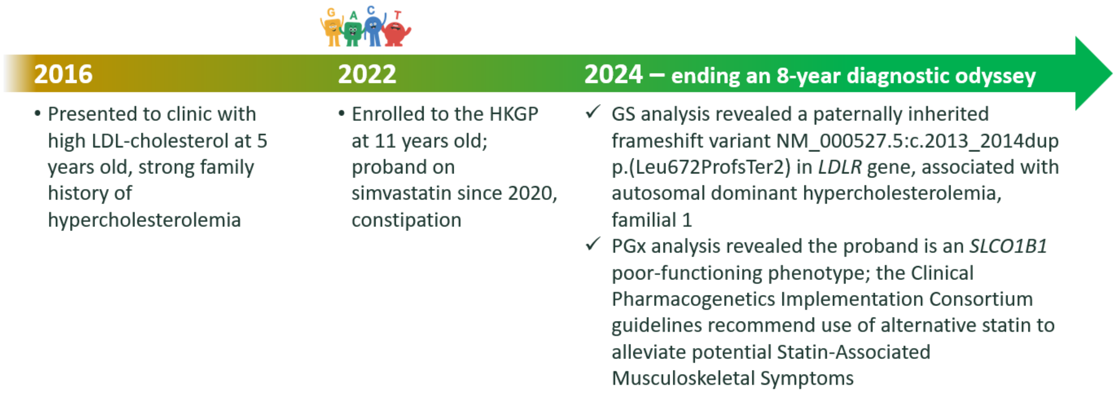

Clinical and genetic evaluation of an 11-year-old male referred for hyperlipidaemia with a strong family history. The proband presented with elevated LDL-cholesterol levels (6.1 mmol/L) at age 5 and has been on simvastatin (20 mg daily) since 2020. Trio-based genome sequencing (GS) identified a heterozygous paternally inherited frameshift variant in the *LDLR* gene (c.2013_2014dup p.(Leu672ProfsTer2)), consistent with a diagnosis of familial hypercholesterolaemia. Pharmacogenomic (PGx) analysis revealed a poor-functioning *SLCO1B1* phenotype (*15/*15), indicating a high risk for statin-associated musculoskeletal symptoms (SAMS), even at low doses of simvastatin, per CPIC guidelines. Symptoms of SAMS range from mild muscle aches to life-threatening rhabdomyolysis; the proband's constipation is suspected to be a manifestation of SAMS. CPIC guidelines recommend an alternative statin to alleviate or prevent SAMS based on the proband's PGx profile. The proband's father, who also has hyperlipidaemia and carries the same *LDLR* variant, is on rosuvastatin and ezetimibe. PGx analysis for the father identified a decreased-functioning *SLCO1B1* phenotype alongside a normally functioning *ABCG2*. CPIC guidelines recommend cautious dosing of rosuvastatin (≤20 mg) to minimize myopathy risk while achieving therapeutic goals.

Supplementary Figure 3: Joint clinical findings for HKGP002040-1

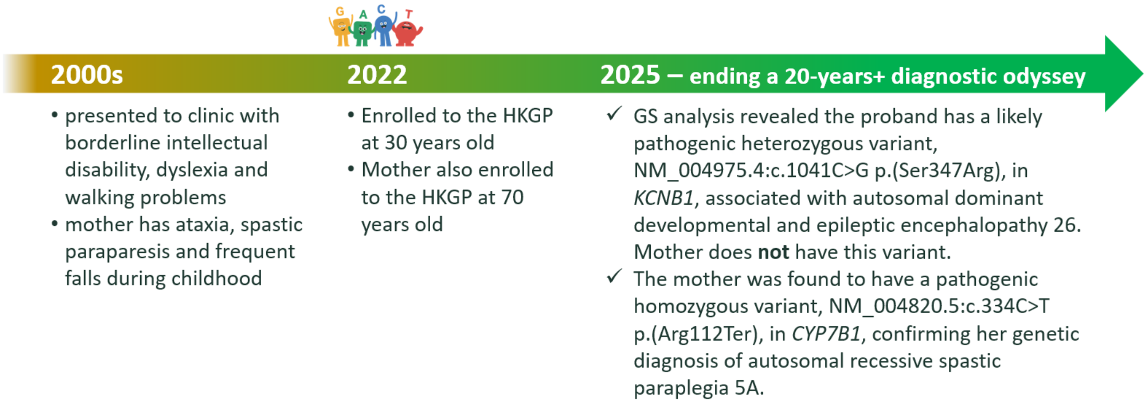

Genetic findings in a 30-year-old male with borderline intellectual disability, dyslexia, and walking difficulties, and his 70-year-old mother, who presented with ataxia, spastic paraparesis, and frequent falls during childhood. Duo genome sequencing (GS) revealed that the proband harboured a likely pathogenic heterozygous variant in the *KCNB1* gene (c.1041C>G p.(Ser347Arg)), consistent with autosomal dominant developmental and epileptic encephalopathy 26 (OMIM: 616056). His mother tested negative for this variant but was found to carry a pathogenic homozygous variant in the *CYP7B1* gene (c.334C>T p.(Arg112Ter)), confirming a diagnosis of autosomal recessive spastic paraplegia 5A (OMIM: 270800).

Supplementary Figure 4: Variant classification method for dominant, recessive and pharmacogenes.

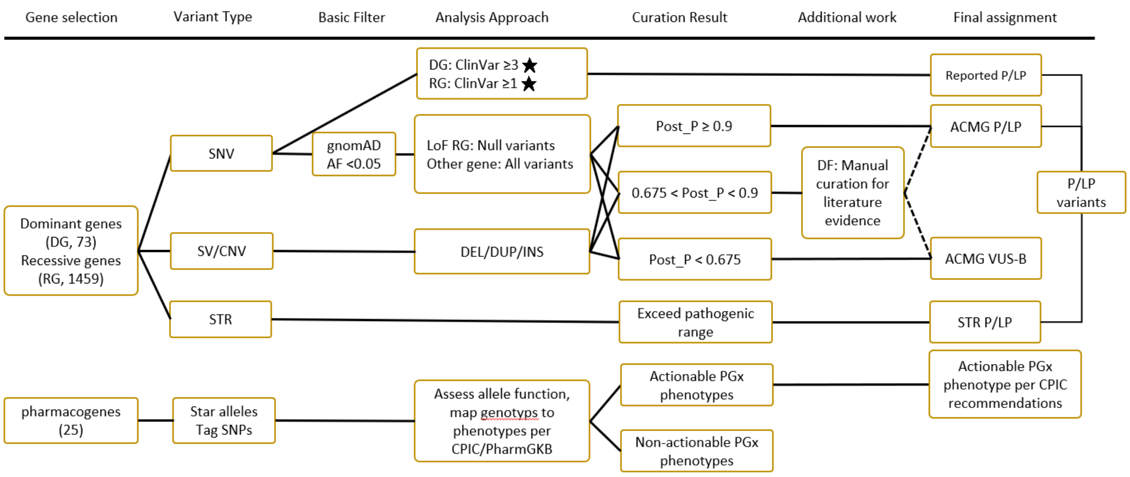

Supplementary Tables

(Table content in supplementary Excel file)

Supplementary Table 1: Full list of probands in the diagnostic cohort, including clinical information and genetic diagnoses.

Supplementary Table 2: Variant details and clinical management of positively diagnosed probands included in the diagnostic cohort.

Supplementary Table 3: Identified pathogenic/likely pathogenic single nucleotide variants (SNVs) and small indels of dominant and recessive genes in the HKGP Chinese cohort.

Supplementary Table 4: Identified Pathogenic/likely pathogenic structural variants, copy number variants and short tandem repeats in dominant and recessive genes in the HKGP Chinese cohort.

Supplementary Table 5: Reclassified pathogenic or likely pathogenic variants in the HKGP Chinese cohort.

Supplementary Table 6: Gene carrier frequency (GCF) of pathogenic or likely pathogenic variants in dominant disorder-related genes.

Supplementary Table 7: Gene carrier frequency (GCF) of pathogenic or likely pathogenic variants and carrier screening tier in recessive disorder-related genes.

Supplementary Table 8: Statistical comparison between two cumulative gene carrier frequency (cGCF) from different populations and Tiering sources.

Supplementary Table 9: Frequencies of altered function alleles for pharmacogenes.

Supplementary Table 10: Number of actionable metabolomic phenotypes for pharmacogenes per participant (source data for Figure 4c).

Supplementary Table 11: Frequency of metabolomic phenotypes for pharmacogenes.

Supplementary Table 12: Top 50 most prescribed drugs in Hong Kong with U.S. Food and Drug Administration (FDA) drug labels and pharmacogenetic associations

Supplementary Table 13: Novel putative protein-disrupting variants in loss-of-function pharmacogenes.

Supplementary Table 14: Novel founder mutations found in the HKGP with shared haplotypes.
