# Supplementary material for: Population-scale genomic medicine with the Hong Kong Genome Project
Source: Nat Med. 2026 May 15;32(6):2277–87. doi: 10.1038/s41591-026-04410-w (PMC13278956; doi:10.1038/s41591-026-04410-w)
Supplement: Supplementary file 2 — Reporting Summary [file 41591_2026_4410_MOESM2_ESM.pdf]

nature portfolio

Corresponding author(s): Brian Hon Yin Chung

Last updated by author(s): Mar 19, 2026

Reporting Summary

Nature Portfolio wishes to improve the reproducibility of the work that we publish. This form provides structure for consistency and transparency in reporting. For further information on Nature Portfolio policies, see our Editorial Policies and the Editorial Policy Checklist.

Statistics

For all statistical analyses, confirm that the following items are present in the figure legend, table legend, main text, or Methods section.

n/a	Confirmed	
☐	☒	The exact sample size (*n*) for each experimental group/condition, given as a discrete number and unit of measurement
☐	☒	A statement on whether measurements were taken from distinct samples or whether the same sample was measured repeatedly
☐	☒	The statistical test(s) used AND whether they are one- or two-sided<br>*Only common tests should be described solely by name; describe more complex techniques in the Methods section.*
☒	☐	A description of all covariates tested
☒	☐	A description of any assumptions or corrections, such as tests of normality and adjustment for multiple comparisons
☒	☐	A full description of the statistical parameters including central tendency (e.g. means) or other basic estimates (e.g. regression coefficient) AND variation (e.g. standard deviation) or associated estimates of uncertainty (e.g. confidence intervals)
☒	☐	For null hypothesis testing, the test statistic (e.g. F, t, r) with confidence intervals, effect sizes, degrees of freedom and P value noted<br>*Give P values as exact values whenever suitable.*
☒	☐	For Bayesian analysis, information on the choice of priors and Markov chain Monte Carlo settings
☒	☐	For hierarchical and complex designs, identification of the appropriate level for tests and full reporting of outcomes
☒	☐	Estimates of effect sizes (e.g. Cohen's d, Pearson's r), indicating how they were calculated

Our web collection on statistics for biologists contains articles on many of the points above.

Software and code

Policy information about availability of computer code

Data collection

No code was used to collect the data analysed in this study.

Data analysis

The code and scripts used to perform all analyses and generate the figures in this study are publicly available on GitHub at: https://github.com/hkgi-steam/hkgi_flagship_paper_2025. The repository includes analysis scripts for identifying variants, generating summary statistics, and producing the display figures. Python 3.10.12 and R 4.1.2 were heavily used in data processing, analysis and visualization. Instructions for reproducing the figures are also provided, including steps to build the required computational environment using Jupyter Notebook and Apptainer.
The following external tools was used for specific analysis:
GATK 4.2.6.1 for variant calling (HaplotypeCaller and Mutect2)
Aldy 4.6 as PGx genotyper
Cyrius 1.1.1 as CYP2D6 caller
HLA-HD 1.7.0 as HLA caller (for HLA-A and HLA-B)
CNVKit 0.9.9 as CNV caller
Manta 1.6.0 as SV caller
ExpansionHunter 3.1.2 STR expansion caller
SNVstory 3.0.2 was employed to infer sub-continental genetic ancestry.
AutoPVS1 2.4 as Classification tool for PVS1 interpretation of null variants
SMNCopyNumberCaller 1.1.2 for SMN genotyping
VEP release 110, GRCh38 for variant annotation

For manuscripts utilizing custom algorithms or software that are central to the research but not yet described in published literature, software must be made available to editors and reviewers. We strongly encourage code deposition in a community repository (e.g. GitHub). See the Nature Portfolio guidelines for submitting code & software for further information.

Data

Policy information about availability of data

All manuscripts must include a data availability statement. This statement should provide the following information, where applicable:

- Accession codes, unique identifiers, or web links for publicly available datasets
- A description of any restrictions on data availability
- For clinical datasets or third party data, please ensure that the statement adheres to our policy

Deidentified proband-level information used for the diagnostic cohort is available in Supplementary Tables 1 and 2. Detailed variant-level information used for the diagnostic cohort and the HKGP Chinese cohort is available in Supplementary Tables 3, 4, 5, 9, 13, and 14. Detailed gene-level information is available in Supplementary Tables 6, 7 and 11. Variants identified in the diagnostic cohort were uploaded to ClinVar in batches (https://www.ncbi.nlm.nih.gov/clinvar/submitters/510250/).
Deidentified individual-level genotype of variants presented in this manuscript, and additional aggregate-level data not included in the manuscript is currently available to researchers upon reasonable request by following these steps:
1. Researchers should submit a Data Access Request to HKGI (hkgi_gc_team@genomics.org.hk) outlining the proposed research, including its purpose, scope of data to be accessed, and researcher information.
2. The HKGI Data Access Review Panel will review the application in a quarterly meeting to assess the scientific, clinical, technical, resource, and regulatory feasibility of the proposal. All feasible proposals will be approved.
3. The HKGI team will collaborate with applicants to prepare the formal proposal and related Institutional Review Board (IRB) documentation.
4. Anonymous, aggregate data will then be provided to applicants either directly or within designated HKGI facilities (for 3-12 months), depending on the assessment of the proposal.
The same application process also applies to other individual-level genomic data beyond this manuscript. As the HKGP is actively recruiting new participants at the time of writing, access to such data will be granted to external researchers after the completion of the main phase of this project in 2030 .

Research involving human participants, their data, or biological material

Policy information about studies with human participants or human data. See also policy information about sex, gender (identity/presentation), and sexual orientation and race, ethnicity and racism.

Reporting on sex and gender	Our findings applied to both sexes, no sex- or gender-based analyses were performed.
Reporting on race, ethnicity, or other socially relevant groupings	We did not analyse social groupings, including race or ethnicity in this study.
Population characteristics	Following characteristics were obtained from the participants in this study:<br>Sex predicted from genomic data analysis,<br>Biological age for sample collection,<br>Health conditions by HPO terms,<br>Ethnicity calculated by genomic data analysis<br>Please refer to Extended Data Table 1 for a breakdown of participant demographics.
Recruitment	For the Hong Kong Genome Project (HKGP), both asymptomatic individuals and symptomatic probands suspected of having a genetic disease were prospectively identified and recruited across a range of medical specialities at the three Partnering Centres of HKGI (the recruitment arm of the HKGP), namely, Hong Kong Children's Hospital, the Chinese University of Hong Kong/Prince of Wales Hospital, and the University of Hong Kong/Queen Mary Hospital. All participants received pretest genetic counselling and provided informed written consent following the unique three-tier consent and assent model designed by the HKGI.
Ethics oversight	Ethics approval was granted by the Central Institutional Review Board (IRB) (HKGP-2021-001, HKGP-2022-001), the IRBs of the Department of Health (L/M257/2021), the Joint Chinese University of Hong Kong-New Territories East Cluster (2021.423, 2023.120), and the University of Hong Kong/Hospital Authority Hong Kong West Cluster (UW 21–413, UW 23–289).

Note that full information on the approval of the study protocol must also be provided in the manuscript.

Field-specific reporting

Please select the one below that is the best fit for your research. If you are not sure, read the appropriate sections before making your selection.

☒ Life sciences ☐ Behavioural & social sciences ☐ Ecological, evolutionary & environmental sciences

For a reference copy of the document with all sections, see nature.com/documents/nr-reporting-summary-flat.pdf

Life sciences study design

All studies must disclose on these points even when the disclosure is negative.

Sample size	The diagnostic cohort (n = 2,227) comprises probands who had completed phenotype-guided diagnostic analysis, supporting personalised genetic diagnosis for individuals with suspected genetic conditions.<br>The HKGP Chinese cohort (n = 18,261) comprises unrelated individuals of Chinese ancestry, selected through stringent relatedness and ethnicity filtering.
Data exclusions	For both cohorts, individuals with sequencing data that failed the quality control were excluded in this study.<br>For HKGP Chinese, participants with sequencing data predicted ethnicity as non-Chinese, or children of other participants were excluded in this study.<br>Using clinical data, we excluded individuals/family members with matching indications from the analysis to minimize sampling bias:<br>- For dominant disorders, 312 participants were excluded due to their phenotypes related to the 73 dominant disorders included in the ACMG secondary findings genes (v3.2).<br>- For recessive disorders, 196 participants were excluded because their children presented primary indications of phenotypes linked to the examined recessive disorder-related genes.<br>- For PGx, 4 participants were excluded because they or their offspring had primary indications matching pharmacogenes with established gene-disease validity.
Replication	Not applicable to this study. For the patient cohort, each individual was treated with a personalized genetic diagnosis; In the Chinese-specific cohort, we eliminated data redundancy to enable more accurate estimation of population frequencies.
Randomization	Not applicable to this study. All comparisons—such as disease or healthy status, pathogenic or benign variants, and increased or normal allele function—were predefined according to public databases and existing guidelines.
Blinding	Not applicable to this study. There was no randomization or intervention. The protocols for sample collections, sequencing, primary and secondary analyses were streamlined.

Reporting for specific materials, systems and methods

We require information from authors about some types of materials, experimental systems and methods used in many studies. Here, indicate whether each material, system or method listed is relevant to your study. If you are not sure if a list item applies to your research, read the appropriate section before selecting a response.

Materials & experimental systems

n/a	Involved in the study	
☒	☐	Antibodies
☒	☐	Eukaryotic cell lines
☒	☐	Palaeontology and archaeology
☒	☐	Animals and other organisms
☐	☒	Clinical data
☒	☐	Dual use research of concern
☒	☐	Plants

Methods

n/a	Involved in the study	
☒	☐	ChIP-seq
☒	☐	Flow cytometry
☒	☐	MRI-based neuroimaging

Clinical data

Policy information about clinical studies

All manuscripts should comply with the ICMJE guidelines for publication of clinical research and a completed CONSORT checklist must be included with all submissions.

Clinical trial registration	Not applicable to this study.
Study protocol	Not applicable to this study.
Data collection	Recruitment started from July 2021 to Nov 2024. And data analysis was carried out from July 2021 to Aug 2025
Outcomes	25% diagnostic yield, 3.8% and 48% carrier rate for dominant and recessive genes. 99.9% for actionable phamacogenomic phenotype rate.

Plants

Seed stocks	Not applicable to this study.
Novel plant genotypes	Not applicable to this study.
Authentication	Not applicable to this study.
