# Supplementary material for: Population-scale genomic medicine with the Hong Kong Genome Project
Source: Nat Med. 2026 May 15;32(6):2277–87. doi: 10.1038/s41591-026-04410-w (PMC13278956; doi:10.1038/s41591-026-04410-w)
Supplement: Supplementary file 3 — Peer Review File [file 41591_2026_4410_MOESM3_ESM.pdf]

nature portfolio

Peer Review File

Population-scale genomic medicine with the Hong Kong Genome Project

Corresponding Author: Dr Brian Hon Yin Chung

This file contains all reviewer reports in order by version, followed by all author rebuttals in order by version.

Version 0:

Reviewer comments:

Reviewer #1

(Remarks to the Author)
Thank you for the opportunity to review this manuscript, describing outcomes from the first 20,000 individuals sequenced through the HK Genome Project. While many national genomic projects publish ambitious plans, manuscripts describing results are few and far between and the HKGP are to be commended on the breadth and quality of their analysis. Undoubtedly, the project will contribute significantly to evidence generation and capacity building in HK, as well as make a major contribution globally.

I have a number of comments/suggestions:
1. Abstract: having read the entire paper, I think perhaps in describing the analysis conducted in asymptomatic individuals, it could be framed as 'screening for medically actionable findings'. I struggled with the use of the term 'carrier' when referring to dominant conditions here and in the Results. The term is usually best reserved for recessive conditions.
2. Introduction. When referring to the work of Australian Genomics, can you please reference the main outcomes paper (PMID 36868206) rather than Mackenzie's Mission? And rephrase to emphasise that evidence generation was the primary objective, which has subsequently led to changes in policy and practice? Similarly, when referring to the work of Genomics England, their other two main outcomes papers, one on the cancer cohort and one on pharmacogenomic findings can be referenced in addition to the outcomes of the RD pilot.
3. Introduction: line 118 "European-centric" does not describe Australian or US studies well. Especially since the goal of AoU is to specifically sequence diverse populations. Consider 'high income countries'? Or 'European ancestries'??
4. Results: individuals enrolled in the project are variably referred to as 'patients', 'cases' and 'probands'. It would be best to pick one term and use it consistently. I would also suggest steering away from any of these terms and using 'individuals' (or 'affected individuals' as needed).
5. Results figures: there are too many different colours used, a different combination in each figure, which is distracting. I would suggest picking a colour scheme and using this throughout. Does the HKGP have its own colour scheme? This would be ideal.
6. Results: I do not see a mention of mitochondrial variants here or in the Methods. Was mitochondrial genome analysis conducted?
7. Results: great data on clinical impact of diagnostic results in the RD cohort. I am unclear as to whether any of the additional medically actionable findings were returned though? And whether you are collecting outcomes data for these? How do you see integration of these within the healthcare system? High volume results such as carrier screening for recessive disorders and pharmacogenomic findings can be particularly challenging. What strategies do you envisage to enable their routine use?
8. Data availability: have the reported variants been submitted to ClinVar? Do you want to comment on whether you are developing infrastructure that will enable the ongoing use of the data generated by HKGP for both clinical care and research. Both Genomics England and AoU provide good examples in this regard. In terms of the reference data generated, is this available to your diagnostic laboratories for example, to improve variant interpretation? Do you plan to contribute to international efforts, e.g. through the network of global gnomADs?
9. Methods: you mention use of PanelApp. Can you please specify whether this is Genomics England PanelApp or PanelApp Australia or both?

Reviewer #2

(Remarks to the Author)
In the Hong Kong Genome Project (HKGP) over 20,000 individuals were sequenced, combining a rare disease cohort with a broader Chinese sample. It reported a 25% diagnostic yield in rare disease patients and developed ancestry-matched references that highlighted shortcomings of European-centric screening panels. While these contributions are important, the project's impact is constrained by its narrow geographical scope. Unlike large-scale initiatives such as the UK Biobank or the U.S. All of Us program, which integrate genomic data with extensive longitudinal and phenotypic records, the HKGP offers little insight into long-term health outcomes or broader population-level trends. The pharmacogenomic analysis reveals Hong Kong-specific alterations in the distribution of different genetic variants where such differences with respect to interethnic variations have been extensively published in previous studies and databases. With respect to other cohorts novel Chinese-specific variants were identified without any important data on altered phenotypes or risks, merely interethnic differences in the distribution of P/LP variants. As a result, the HKGP represents a valuable starting point for Hong Kong-specific genomic resources but falls short of delivering novel holistic genotype–phenotype links and global comparability achieved by other major international efforts.

The study suggests that population-level knowledge of genetic variants can enhance clinical diagnosis and treatment. While recognizing the importance of identifying interregional differences, such insights do not automatically translate into better care or more effective drug therapy for individual patients. In practice, whole-genome sequencing (WGS) is expected to become increasingly central to personalized medicine, particularly in oncology and eventually as part of routine clinical practice. Regarding genetic variants specific for tumors the WGS technique increases in application for identification of the causal variants. For these purposes, the global distribution of variants is less relevant than the predictive power of specific genetic variants for improving drug treatment in individual cases.

Relatively little emphasis is given to relevant previous studies in the field encompassing very large Chinese populations such as PMID: 40634300, PMID: 39991480 and the China Kadoorie Biobank (CKB) genetic resources.

In the pharmacogenomic section, the authors group variants across multiple genes—such as ADME genes involved in drug response and toxicity—but provide little discussion of the relative clinical significance of the specific variants. The impact of different genetic variants on clinical outcomes varies considerably. Broader approaches, such as the European PREPARE study (>7,000 patients), did not demonstrate a reduction in drug-induced adverse reactions through predictive genotyping, largely due to the low prevalence of actionable variants, the important placebo effects and the absence of a targeted focus on specific gene–drug pairs (PMID: 40552566). In contrast, the most informative insights typically emerge from analyses of well-defined gene–drug pairs. This is underscored by the FDA's compilation of pharmacogenomic biomarkers in drug labeling, which highlights the relative importance of specific gene–drug pairs across therapeutic areas (see https://www.fda.gov/drugs/science-and-research-drugs/table-pharmacogenomic-biomarkers-drug-labeling and https://www.fda.gov/medical-devices/precision-medicine/table-pharmacogenetic-associations. Thus, the authors provide a list (Table 2) of the most commonly prescribed drugs in Hong Kong, linked to genes with actionable variants, but they do not connect this to population-specific genetic variants that could significantly influence drug response and side-effect profiles. In addition, when compared with the FDA's classification of gene–drug pairs by clinical importance in pharmacogenomics—specifically those listed in the Table of Pharmacogenetic Associations for which the Data Support Therapeutic Management Recommendations (https://www.fda.gov/medical-devices/precision-medicine/table-pharmacogenetic-associations), the overlap is limited. Of the 20 most frequently prescribed drugs in Hong Kong with actionable pharmacogenomic phenotypes (Table 2), only 3 are recognized by the FDA as having sufficient evidence to support therapeutic management recommendations.

Other comments
• Since all individuals were recruited based on suspicion of having a genetic disease, it is not appropriate to consider them as a general disease cohort. They are clearly distinct from the broader population with respect to germline variation.
• Some pharmacogenes have endogenous substrates (e.g., ABCG2) and are linked to congenital diseases. The cohort may therefore be enriched for such mutations, and this potential bias should be considered in the pharmacogenomic profiling.
• Figure 1a could be reordered—by either sample size or diagnostic yield—to present the message more clearly.
• In Figure 4a, it appears that each gene was compared to a single CPIC reference population. How was this reference population selected?
• In Table 1, the first category should be the 553 patients with positive genetic diagnoses, rather than the entire diagnostic cohort.

Reviewer #4

(Remarks to the Author)
The article entitled "Bridging the Genomic Gap: The Hong Kong Genome Project's Initiative in Unlocking Population-tailored Genomic Medicine" by Ying et al describes a flagship study highlighting the enormous undertaking of precision medicine initiatives when it comes to analysis of individual datasets across 20,000 participants and collectively looking at the actionable impact at the level of the population. The HKGP achieved significant outcomes: a diagnostic yield of 25% (553/2,227), clinical utility of 88% for patients across diverse disease categories, identification of clinically actionable findings in 564 dominant disorder-related genes in 3.7% (670/17,949) of participants for personalised risk assessment, carrier status for ACMG-listed recessive disorder genes in 48% (8,693/18,065) of participants, and actionable pharmacogenomic phenotypes in nearly all participants, with potential to inform more than 0.9 million prescriptions annually in Hong Kong. Importantly, they established a population-specific genomic reference dataset, addressing a critical knowledge gap in Asian genomic data. The authors should be commended for their high-quality work. I have no major concerns but have a few comments to improve clarity in a few sections.

Comments:
1. Were results returned to all participants, and if so how? I recognized that the rare disease diagnoses were likely returned to the clinicians at the respective hospitals, but what about the carrier screening outcomes for example. How was education of participants supported so they could understand their results? Can you add more details or a section addressing this in the methods as returning this number of actionable results is no minor undertaking.
2. Similarly, how did you deal with clinically actionable findings that were not related to the referral indication for children and youth - so the 564 dominant disorder genes and the ACMG-listed recessive disorder genes – were these returned even if not actionable in childhood? Was there a choice about being informed about these risks for those under 18 years of age?
3. Can you comment on the use of buccal/saliva? How many of the samples were buccal/saliva, what was the quality? In my experience, buccal samples for genomes tend to be messy and require much higher depth of coverage, especially for the identification of de novo variants.
4. On line 868, it states that orthogonal validation was performed for P/LP diagnostic findings – can you expand on this in the methods? Does that mean that all SNVs, CNVs, STRs etc were confirmed in a secondary assay? With a new sample from the participant? In particular, in my experience bioinformatic tools such as Expansion Hunter aren't yet reliable in calling STRs, especially with short read data, so secondary testing would be important to confirm expansion size.
5. On line 870, it states that VUS were reported only if they were clinically actionable. Does this mean that only if a secondary assay, like enzyme or biomarker testing, could confirm the diagnosis? Please expand.
6. How is the HKGP contributing to global datasets like gnomad and ClinVar so that all Asian individuals can benefit no matter where they live in the world?
7. The 17.5% of diagnosed patients who became clinical trial eligible seems high (Figure 1). Can you expand on what types of trials they were eligible for as my review of the diagnostic details suggests there are many different diseases captured in that percentage.
8. While high altered-function allele frequencies—such as those exceeding 10% for ABCG2, CYP2B6, UGT1A1, HLA-B, SLCO1B1, and CYP2D6—may suggest population-level relevance, AMP's criteria for reportable pharmacogenes are based on clinical actionability, not frequency alone. This would be important to keep in mind because how would a region tailor pharmaceutical dosing for 0.9M people; definitely challenges with scaling here.

Editorial:
1. Line 182: 'Demographics and clinical characteristics OF the diagnostic cohort'
2. Fig 1b, please indicate that the total number who received a diagnosis was 553
3. Suppl Table 9 – 'DecreasEd metabolism of nicotine'

Version 1:

Reviewer comments:

Reviewer #1

(Remarks to the Author)
Thank you for the opportunity to review a revised version of this manuscript.

All of my previous comments have been addressed well.

I would still strongly encourage the authors to make a large submission to ClinVar as part of this publication, even if it is not for all the variants.

Reviewer #2

(Remarks to the Author)
Reviewer 2 Comments to rebuttal

Reviewer #2 Comment 1
In the Hong Kong Genome Project (HKGP) over 20,000 individuals were sequenced, combining a rare disease cohort with a broader Chinese sample. It reported a 25% diagnostic yield in rare disease patients and developed ancestry-matched references that highlighted shortcomings of European-centric screening panels. While these contributions are important, the project's impact is constrained by its narrow geographical scope. Unlike large-scale initiatives such as the UK Biobank or the U.S. All of Us program, which integrate genomic data with extensive longitudinal and phenotypic records, the HKGP offers little insight into long-term health outcomes or broader population-level trends. The pharmacogenomic analysis reveals Hong Kong-specific alterations in the distribution of different genetic variants where such differences with respect to interethnic variations have been extensively published in previous studies and databases. With respect to other cohorts novel Chinese-specific variants were identified without any important data on altered phenotypes or risks, merely interethnic differences in the distribution of P/LP variants. As a result, the HKGP represents a

valuable starting point for Hong Kong-specific genomic resources but falls short of
delivering novel holistic genotype–phenotype links and global comparability achieved
by other major international efforts.

Reviewers new response
In the authors' response, no new information is provided that would alter the original statement that this study is geographically restricted and lacks novel holistic genotype–phenotype links and that this kind of investigation have previously done in major international projects.

Reviewer #2 Comment 2
The study suggests that population-level knowledge of genetic variants can enhance clinical diagnosis and treatment. While recognizing the importance of identifying interregional differences, such insights do not automatically translate into better care or more effective drug therapy for individual patients. In practice, whole-genome sequencing (WGS) is expected to become increasingly central to personalised medicine, particularly in oncology and eventually as part of routine clinical practice. Regarding genetic variants specific for tumors the WGS technique increases in application for identification of the causal variants. For these purposes, the global distribution of variants is less relevant than the predictive power of specific genetic variants for improving drug treatment in individual cases.

Reviewers new response

A summary of the reviewers statement here is that the reviewer is sceptical that population- or geography-based variant data meaningfully improves individual patient care and argues that clinically actionable, predictive genetic information obtained through WGS is more important than knowing how variants are distributed across populations. This criticism is still valid

Reviewer #2 Comment 3
Relatively little emphasis is given to relevant previous studies in the field encompassing very large Chinese populations such as PMID: 40634300, PMID: 39991480 and the China Kadoorie Biobank (CKB) genetic resources.

Reviewers new response

In contrast to the authors' response, the reviewer considers these large studies to be highly important and believes they should be discussed in the manuscript, including differences in methodology. However, the authors have chosen not to address them.

Reviewer #2 Comment 4
In the pharmacogenomic section, the authors group variants across multiple genes such as ADME genes involved in drug response and toxicity but provide little discussion of the relative clinical significance of the specific variants. The impact of different genetic variants on clinical outcomes varies considerably. Broader approaches, such as the European PREPARE study (>7,000 patients), did not demonstrate a reduction in drug induced adverse reactions through predictive genotyping, largely due to the low prevalence of actionable variants, the important placebo effects and the absence of a targeted focus on specific gene–drug pairs (PMID: 40552566). In contrast, the most informative insights typically emerge from analyses of well-defined gene–drug pairs. This is underscored by the FDA's compilation of pharmacogenomic biomarkers in drug labelling, which highlights the relative importance of specific gene–drug pairs across therapeutic areas (see https://www.fda.gov/drugs/science-and-research-drugs/tablepharmacogenomic-biomarkers-drug-labeling and https://www.fda.gov/medicaldevices/precision-medicine/table-pharmacogenetic-associations. Thus, the authors provide a list (Table 2) of the most commonly prescribed drugs in Hong Kong, linked to genes with actionable variants, but they do not connect this to population-specific genetic variants that could significantly influence drug response and side-effect profiles.
In addition, when compared with the FDA's classification of gene–drug pairs by clinical importance in pharmacogenomics—specifically those listed in the Table of Pharmacogenetic Associations for which the Data Support Therapeutic Management Recommendations(https://www.fda.gov/medical-devices/precision-medicine/tablepharmacogenetic-associations), the overlap is limited. Of the 20 most frequently prescribed drugs in Hong Kong with actionable pharmacogenomic phenotypes (Table 2), only 3 are recognized by the FDA as having sufficient evidence to support therapeutic management recommendations.

Reviewers new response
In summary, the reviewer argues that the pharmacogenomic analysis is overly broad and insufficiently grounded in clinically validated gene–drug relationships. The reviewer thinks that the study overstates the clinical relevance of its findings. Although the authors present a list of commonly prescribed drugs in Hong Kong linked to genes with actionable variants, they fail to connect these drugs to population-specific genetic variants that could meaningfully influence drug response or adverse-effect profiles. Consequently, the reviewer concludes that the analysis lacks population-specific actionable insights and does not align well with established regulatory evidence for pharmacogenomic utility.

Reviewer #2 Comment 5
Since all individuals were recruited based on suspicion of having a genetic disease, it is not appropriate to consider them as a general disease cohort. They are clearly distinct from the broader population with respect to germline variation.

Reviewers new response:
The authors have now clarified the recruitment strategy, cohort composition, and analytical approach.

Reviewer #2 Comment 6
Some pharmacogenes have endogenous substrates (e.g., ABCG2) and are linked to congenital diseases. The cohort may therefore be enriched for such mutations, and this potential bias should be considered in the pharmacogenomic profiling.

Reviewers new comments
The authors have satisfactory dealt with this point.

Reviewer #2 Comment 7
Figure 1a could be reordered—by either sample size or diagnostic yield—to present the message more clearly.

Reviewers new comment: This has been satisfactory dealt with

.
Reviewer #2 Comment 8
In Figure 4a, it appears that each gene was compared to a single CPIC reference population. How was this reference population selected?

Reviewers new comment: This has been satisfactory dealt with

Reviewer #2 Comment 9
In Table 1, the first category should be the 553 patients with positive genetic diagnoses, rather than the entire diagnostic cohort.

Reviewers new comment: This has been satisfactory dealt with

Reviewer #4

(Remarks to the Author)
Thank you to the authors for their thoughtful responses to my previous comments. I appreciated the data availability update; from your responses, it looks like you will not be contributing the aggregate allele frequency data to gnomad at this time but will submit the P/LP variants to ClinVar. I also appreciated the clarity on which/how results were being returned at this stage of the program and recognize that the program needs to understand the scale of the challenge before secondary findings, carrier results, and pharmacogenomics are returned at scale.

Minor comments:

Line 939: edits to: “VUSs in dominant genes that meet following criteria, agreed upon by all parties in the multidisciplinary team (MDT), including clinicians, were reported: highly compatible with the clinical phenotypes, and when additional secondary assay/analysis—such as RNA sequencing, enzyme activity testing, immunohistochemical staining, imaging studies, and segregation analysis, can be performed to confirm the diagnosis.”

Line 267: re: “Among the 97 probands eligible to clinical trials (17.5%), 75 (13.6%) were eligible for interventional clinical trials, with 9 of these involving phase III/IV trials, while 22 (3.9%) were eligible for observational clinical trials.” GJB2-related hearing loss and NF1 account for 50 patients of the 97 – please add this detail so that no one thinks it is 97 different trials.

This section needs an edit for repeating words/phrases (page 6, line 164-175, in Introduction) – see suggestions here: “The integrated results derived from the project’s data involve four key components and pursue four core aims: (1)

characterising population-wide diagnostic genomic variation in Hong Kong; (2) enabling early intervention and preventive care for asymptomatic individuals through an analysis of pathogenic or likely pathogenic (P/LP) variants in genes associated with dominant disorders, such as tumour syndromes and cardiovascular diseases; (3) informing reproductive planning through an analysis of carrier frequencies for recessive genetic disorders; and (4) optimising treatment efficacy, reducing adverse drug reactions, and improving therapeutic outcomes through pharmacogenomic profiling."

Open Access This Peer Review File is licensed under a Creative Commons Attribution 4.0 International License, which permits use, sharing, adaptation, distribution and reproduction in any medium or format, as long as you give appropriate credit to the original author(s) and the source, provide a link to the Creative Commons license, and indicate if changes were made.
In cases where reviewers are anonymous, credit should be given to 'Anonymous Referee' and the source.
The images or other third party material in this Peer Review File are included in the article's Creative Commons license, unless indicated otherwise in a credit line to the material. If material is not included in the article's Creative Commons license and your intended use is not permitted by statutory regulation or exceeds the permitted use, you will need to obtain permission directly from the copyright holder.
To view a copy of this license, visit https://creativecommons.org/licenses/by/4.0/

Reviewer #1 Comment 1

Abstract: having read the entire paper, I think perhaps in describing the analysis conducted in asymptomatic individuals, it could be framed as 'screening for medically actionable findings'. I struggled with the use of the term 'carrier' when referring to dominant conditions here and in the Results. The term is usually best reserved for recessive conditions.

Response:

Thank you for your insightful feedback, which helps improve the clarity and precision of the manuscript. We have implemented the suggested changes throughout the manuscript. Particularly, in the Abstract, we revised the description to:

> *"Our Chinese-specific cohort (n=18,261) underwent genomic screening for medically actionable findings."* (page 4, line 82-83, in **Abstract**)

Additionally, recognising that the term 'carrier' should be reserved for recessive conditions, we have amended the text accordingly. The Abstract now states:

> *"the analysis revealed that 3.7% of the individuals had pathogenic or likely pathogenic variants associated with dominant disorders."* (page 4, line 84-85, in **Abstract**)

To avoid confusion, we have replaced the "carrier frequency" to "prevalence" in the subheading of the results associated with dominant disorder-related genes, and it now reads:

> *"Prevalence of clinically actionable variants in dominant disorder-related genes"* (page 13, line 339, in **Results**)

Reviewer #1 Comment 2

Introduction. When referring to the work of Australian Genomics, can you please reference the main outcomes paper (PMID 36868206) rather than Mackenzie's Mission? And rephrase to emphasise that evidence generation was the primary objective, which has subsequently led to changes in policy and practice? Similarly, when referring to the work of Genomics England, their other two main outcomes papers, one on the cancer cohort and one on pharmacogenomic findings can be referenced in addition to the outcomes of the RD pilot.

Response

Thank you for this suggestion. The reference to Mackenzie's Mission has been replaced with the main outcomes paper of Australian Genomics (PMID 36868206)[5] . The text has also been revised to emphasise the primary objective, which was evidence generation

that has subsequently led to changes in policy and practice. For Genomics England, references to the two additional outcomes papers (the cancer cohort PMID: 38200255; pharmacogenomic findings PMID: 39481076)[6,7] have been included alongside the outcomes of the RD pilot.

The Introduction has been updated accordingly:

> "*For diagnostic purposes, the 100,000 Genomes Project in the UK achieved a diagnostic yield of 25% for RDs before the implementation of genome sequencing (GS) by the National Health Service (NHS)*[2]*, and expand the outcome study on cancer and pharmacogenomics*[3,4]*. While Australian Genomics primarily focused on evidence generation, this work had subsequently led to changes in policy and practice to improve the equity of access to diagnostic testing*[5]*."* (page 5, line 111-116, in **Introduction**)

Reviewer #1 Comment 3

Introduction: line 118 "European-centric" does not describe Australian or US studies well. Especially since the goal of AoU is to specifically sequence diverse populations. Consider 'high income countries'? Or 'European ancestries'??

Response:

Thank you for pointing this out. We agree that the revision would ensure greater clarity and accuracy in describing the studies referenced, as the term 'European-centric' may not accurately describe Australian or US studies, particularly initiatives like the All of Us (AoU) program, which aim to sequence diverse populations. To address this, we have revised the text to use 'European ancestry' where appropriate. For example, we now refer to 'European ancestry-based panels' and large-scale initiatives focusing on populations of European ancestry.' In the Abstract and Introduction, respectively:

> "*By streamlining genetic screening using European-ancestry-based panels"* (page 4, line 83-84, in **Abstract**), and
>
> *"In addition to these large-scale initiatives based upon European ancestries"* (page 5, line 120-121, in Introduction)

And other multiple sentences in the **Results.**

Reviewer #1 Comment 4

Results: individuals enrolled in the project are variably referred to as 'patients', 'cases 'and 'probands'. It would be best to pick one term and use it consistently. I would also suggest steering away from any of these terms and using 'individuals' (or 'affected individuals' as needed).

Response:

Thank you for pointing out this inconsistency. We have revised the manuscript to ensure consistent terminology throughout. The term 'probands' is now used exclusively for the analysis of the diagnostic cohort, unless specific distinctions are required. This change has been applied consistently across the manuscript to improve clarity and alignment with your suggestion.

Reviewer #1 Comment 5

Results figures: there are too many different colours used, a different combination in each figure, which is distracting. I would suggest picking a colour scheme and using this throughout. Does the HKGP have its own colour scheme? This would be ideal.

Response:

Thank you for your comment. We have revised the colour schemes across all figures to ensure consistency and clarity. Specifically, we have adopted the colour scheme used in **Figure 3** (recessive genes) for other figures (1,2,4) throughout the manuscript.

Reviewer #1 Comment 6
Results: I do not see a mention of mitochondrial variants here or in the Methods. Was mitochondrial genome analysis conducted?

Response:

We thank the reviewer for raising this important point regarding mitochondrial variants. We confirm that mitochondrial genome analysis was well covered in this study, and have added the detection and analysis in the Methods section:

> *"mitochondrial genome was performed using GATK HaplotypeCaller and Mutect2 (v4.2.6.1)"* (page 3, line 875-876, in **Methods**), and
>
> *"Specifically, mitochondrial variants were analysed according to the ClinGen Mitochondrial Disease Nuclear and Mitochondrial Expert Panel Specifications to the ACMG/AMP Variant Interpretation Guidelines."* (page 34, line 917-919, in **Methods**)

As a result, we detected 2 pathogenic mitochondria variants in our diagnostic cohort (see table below, extracted from **Supplementary Table 2**). In addition, *MT-RNR1* was included in our pharmacogenomic screening panel, where we observed alternative alleles in the 4 tag SNPs in the gene among 109 out of 18,257 individuals (**Supplementary Table 9**).

HKGP ID	Gene	Variant detected	Heteroplasmy (in blood)	Diagnosis

HKGP000096-1	MT-ATP6	NC_012920.1:m.9185T>C p.(Leu220Pro)	85%	Mitochondrial DNA-Associated Leigh Syndrome and NARP (OMIM: 256000)
HKGP001923-1	MT-ATP6	NC_012920.1:m.8993T>C p.(Leu156Pro)	98%	Mitochondrial DNA-Associated Leigh Syndrome and NARP (OMIM: 256000)

Reviewer #1 Comment 7

Results: great data on clinical impact of diagnostic results in the RD cohort. I am unclear as to whether any of the additional medically actionable findings were returned though? And whether you are collecting outcomes data for these? How do you see integration of these within the healthcare system? High volume results such as carrier screening for recessive disorders and pharmacogenomic findings can be particularly challenging. What strategies do you envisage to enable their routine use?

Response:

Thank you for the comment. We have expanded the section to address findings disclosure, particularly noting:

> *"Three years post-pilot, HKGP stands at a critical juncture in genomic findings disclosure. While our current protocol returns only primary findings, it was developed with careful consideration of key stakeholders' preferences[35], and this study serves as the first step toward broader return options."* (page 27, line 657-661, in **Discussion**)

We have also added the details on reporting and returning results in the **Supplementary Information** (page 3, line 73-97) as follows:

> ***Primary Findings***
> *Building on patient and clinician feedback, we will continue to prioritise returning clinically significant findings directly related to the referral indication and clinical phenotype.*
>
> ***Additional medically actionable findings***
> ***Dominant Disorders:*** *For participants opt-in for feedback of additional findings of GS, we have developed a plan for reporting and returning findings in 13 genes (of which, 12 are associated with dominant disorders) – MLH1; MSH2; MSH6; MUTYH; APC; BRCA1; BRCA2; VHL; MEN1; RET; LDLR; APOB; PCSK9 – based on clinical actionability and severity. The gene list will be reviewed and revised regularly, by the Scientific and Ethics Advisory Committees of HKGI, to align with evolving international standards. In compliance with ACMG guidelines and reporting guidance, only pathogenic or likely pathogenic variants will be reported* (https://search.clinicalgenome.org/kb/genes/acmgsf)*. Specific conditions apply:*

for child participants, only genes associated with childhood-onset diseases will be returned. This structured approach ensures responsible return of high-impact genetic information while respecting clinical context and participant preferences.
Recessive Disorders: *For reporting and returning additional findings of MUTYH-associated polyposis, only individuals with two identified disease-causing variants will receive results. Regarding expanded carrier screening, we are at the crossroads. While we will continue to return carrier status upon patient request, this study reinforces our decision to develop a Chinese-specific carrier screening panel rather than relying solely on resources based on European ancestries like ACMG and Mackenzie's Mission. We have demonstrated our capability to identify and return these results to patients.*

We are actively collecting multidimensional outcomes data through an HKGP evaluation framework. While existing research on the clinical utility of genome sequencing often relies on simplistic metrics—such as the percentage of individuals with altered healthcare management—these approaches may fail to capture the full spectrum of impacts. Our HKGP evaluation framework addresses this gap through a singular, validated metric to evaluate clinical utility. Our unpublished study found that GS not only had impact on traditional clinical utility metrics (e.g., referrals for surveillance and medication changes) but also profoundly affects psychological health by offering clarity, empowerment, and hope, thereby reducing years of uncertainty and anxiety. Furthermore, excluding certain genetic conditions provides significant reassurance, alleviating ongoing health concerns and fostering peace of mind. This comprehensive evaluation reveals that the true clinical utility of GS extends beyond healthcare management changes to encompass substantial psychosocial benefits, providing a more nuanced understanding of its value for patients and healthcare systems.
We recognise that high-volume genomic results, such as carrier screening for recessive disorders and pharmacogenomic findings, present unique challenges for routine clinical implementation. As part of our ongoing protocol evolution, we are actively developing strategies to address these challenges through multidisciplinary review by our Scientific and Ethics Advisory Committee and other stakeholders. This process will incorporate population-specific insights from our HKGP findings—including the development of a Chinese-specific carrier screening panel—and consider optimal pathways for delivering pharmacogenomic results. Our approach will balance clinical utility, resource efficiency, and ethical considerations to enable responsible integration of these findings into healthcare.

Reviewer #1 Comment 8

Data availability: have the reported variants been submitted to ClinVar? Do you want to comment on whether you are developing infrastructure that will enable the ongoing use of the data generated by HKGP for both clinical care and research. Both Genomics England and AoU provide good examples in this regard. In terms of the reference data

generated, is this available to your diagnostic laboratories for example, to improve variant interpretation? Do you plan to contribute to international efforts, e.g. through the network of global gnomADs?

Response

HKGI has engaged with leaders from various genome institutes and genome projects, including the Hospital for Sick Children (Canada), Harvard Medical School (USA), Australian Genomics, Genomics England and other universities, to explore the clinical, personal, and system-wide value of genomic medicine. We contributed to

1. Defining utility metrics: Advancing tools like the GUV Scale (Genomic Utility Value) for policy decisions (PMID: 41310283)[9]
2. Global data sharing: Supporting GA4GH standards to scale genomic data interoperability across health systems.

HKGP has a strategic plan in place to contribute to global datasets such as ClinVar, ensuring that Asian individuals can benefit from these resources regardless of where they live. Specifically:

1. Contribution Strategy: Variants classified as pathogenic or likely pathogenic are released to clinicians following diagnostic reporting. Updates or additional feedback on the pathogenicity of these variants will be incorporated within 6 months. After this process, the variants will be summarised for publication and submitted to ClinVar to ensure they are shared with the broader scientific and clinical community.
2. Progress to Date: As part of this strategy, the first batch of 45 variants identified in our pilot study, published earlier this year and also included in the diagnostic cohort in this study, has already been uploaded to ClinVar. (Submitter: Hong Kong Genome Institute https://www.ncbi.nlm.nih.gov/clinvar/submitters/510250/)
3. Future Contributions: For the diagnostic cohort, which includes 573 variants, we plan to follow the same protocol. These variants, as well as other newly curated positive variants beyond this study, will be uploaded to ClinVar or other related public repositories in batches, ensuring timely sharing of valuable genomic insights.

This approach underscores HKGP's commitment to advancing the representation of Asian populations in global genomic datasets and fostering equitable access to genomic knowledge worldwide. **Supplementary Table 2** that listing all positive variants found in the diagnostic cohort, was updated by adding the ClinVar submitter URL.

Reviewer #1 Comment 9

Methods: you mention use of PanelApp. Can you please specify whether this is Genomics England PanelApp or PanelApp Australia or both?

Response:

Thank you for pointing this out. We confirm that both Genomics England PanelApp and PanelApp Australia were used to make the screening more comprehensive. This has been clarified in the Methods section of the manuscript:

> *"Genes with strong or definitive gene–disease associations, as classified by ClinGen ("definitive" or "strong") and Genomics England PanelApp and PanelApp Australia ("green"), were prioritised."* (page 33, line 882 – 884, in **Methods**)

Reviewer #2 Comment 1

In the Hong Kong Genome Project (HKGP) over 20,000 individuals were sequenced, combining a rare disease cohort with a broader Chinese sample. It reported a 25% diagnostic yield in rare disease patients and developed ancestry-matched references that highlighted shortcomings of European-centric screening panels. While these contributions are important, the project's impact is constrained by its narrow geographical scope. Unlike large-scale initiatives such as the UK Biobank or the U.S. All of Us program, which integrate genomic data with extensive longitudinal and phenotypic records, the HKGP offers **little insight into long-term health outcomes or broader population-level trends.** The pharmacogenomic analysis reveals Hong Kong-specific alterations in the distribution of different genetic variants where such differences with respect to interethnic variations have been extensively published in previous studies and databases. With respect to other cohorts novel Chinese-specific variants were identified without any important data on altered phenotypes or risks, merely interethnic differences in the distribution of P/LP variants. As a result, the HKGP represents a valuable starting point for Hong Kong-specific genomic resources but **falls short of delivering novel holistic genotype–phenotype links** and global comparability achieved by other major international efforts.

Response:

Thank you for your thoughtful comments regarding the scope and contributions of the Hong Kong Genome Project (HKGP). We appreciate the opportunity to clarify the project's scope and highlight the novel findings included in our revision. The HKGP is a clinically oriented project with aims that extend beyond individual-level benefits to population-level impacts. This study provided an interim summary of the findings generated by our established database. In particular, we enriched and restructured the aims of the project and the current study:

> "*Led by the Hong Kong Genome Institute (HKGI), which was established in May 2020 as a wholly government-owned entity, the HKGP, which is the first and largest GS initiative in Hong Kong, aims to integrate genomics into clinical care, advance genomic research, enhance genomic literacy, and cultivate special talent.*" (page 6, line 144-148, in **Introduction**), and

> "*The integrated results derived from the project's data involve four key components and pursue four core aims: (1) characterising population-wide genomic variation with the diagnostic genetic variant landscape that characterises population-wide genomic variation in Hong Kong; (2) enabling early intervention and preventive care for asymptomatic individuals through an analysis of pathogenic or likely pathogenic (P/LP) variants in genes associated with dominant disorders, such as tumour syndromes and cardiovascular diseases, to enable early intervention and preventive care for asymptomatic individuals; (3) informing reproductive planning through an analysis of carrier frequencies for recessive genetic disorders to inform reproductive planning; and (4) optimising treatment*

efficacy, reducing adverse drug reactions, and improving therapeutic outcomes through pharmacogenomic profiling and implications from the analysis of drug response alleles." (page 6-7, line 164-175, in **Introduction**).

Unlike population biobanks mainly focusing on population level impact (e.g., UK Biobank, All of Us), HKGP drives genomic discoveries directly into clinical pathways, positioning it alongside transformative clinical implementations like the 100,000 Genomes Project in the UK.

Building on this clinical foundation, HKGP's impact is multifaceted. Not only does it address population-level biases in ACMG and CPIC gene panels, but it has also enabled novel variant discovery by identifying and reclassifying previously unreported pathogenic variants in our diagnostic cohort and the HKGP Chinese cohort for clinically actionable variants in dominant/recessive disorder-related genes (**Table 1** for summary**,** and **Supplementary Table 2, 5** for more details). With the comprehensive WGS approach, we extended our analysis to cover novel PGx discovery (**Supplementary Table 13**). These advances have collectively facilitated diagnoses and preventive care for participants.

Particularly in our pharmacogenomics (PGx) analysis, we focused on actionable alleles with clinical significance, as defined by CPIC guidelines. In response to the reviewer's valuable **Comment 4** below, we have expanded this analysis to also incorporate relevant FDA guidelines. This revised section now addresses actionable alleles based on both CPIC and FDA recommendations. This additional analysis revealed that 2.8 out of 5.2 actionable phenotypes are associated with high clinical impact according to FDA PGx therapeutic recommendations. This comprehensive WGS approach also enabled novel PGx discovery:

"Novel variants in loss-of-function pharmacogenes

To further investigate novel deleterious variants in pharmacogenes, SNVs, small indels, CNVs and SVs were detected using the same methodology described earlier. This analysis focused on nine pharmacogenes for which no-function alleles have been defined to be associated with actionable phenotype by CPIC or PharmGKB (CYP2B6, CYP2C9, CYP2C19, CYP2D6, DPYD, G6PD, NUDT15, SLCO1B1 and TPMT). These genes were selected based on the rationale that LoF is a mechanism associated with their actionable phenotype. Only putative protein-disrupting variants, including frameshift, inframe, splicing, and nonsense variants in these genes with PVS1 strength reach "Very Strong" from AutoPVS1 were included in this study after manual investigation on IGV for to ensure high quality variants." (page 40, line 1120-1130, in **Methods**)

"Novel variants in loss-of-function pharmacogenes

To evaluate potentially deleterious novel pharmacogenetic variants, we identified putative protein-disrupting variants in 9 of the 25 pharmacogenes, where loss-of-function (LoF) is the mechanism associated with the actionable phenotype. A total of 108 variants were detected in 8 out of 10 genes from 340 (1.86%)

individuals, including 30 SNVs, 63 small indels, and 15 large deletions and duplications. Among these, 88 (81.5%) variants were absent in gnomAD. Specifically, 81 (75.0%) variants were unique to single individuals, suggesting a high degree of individual specificity. Notably, 70 (64.8%) variants were found in DPYD, SLCO1B1, and G6PD, indicating that these genes may harbour a particularly high burden of novel pharmacogenetic variants (Supplementary Table 13). The high prevalence of rare risk and putative protein-disrupting variants in pharmacogenes, including large deletions and insertions, highlights the need for WGS in comprehensive pharmacogenetic testing, as genotyping assays may miss or misidentify these variants." (page 22, line 562-575, in **Results**)

Besides novel discovery, the high frequency of rare and potentially disruptive variants in pharmacogenes highlights HKGP's value as a starting point for developing population-specific pharmacogenomic guidance. While this study does not assess long-term clinical outcomes, our linked health records provide a foundation for forthcoming longitudinal analyses. These efforts will define the real-world health impacts of these variants, advancing precision medicine in our community.

This new evidence, together with the novel findings in dominant and recessive genes, underscores the primary novelty of the project—the generation of a high-resolution, WGS dataset for a historically underrepresented population. While we concur that documenting Hong Kong Chinese-specific distributions of known variants is an important outcome, the project's deeper contribution, as now demonstrated with this new analysis, lies in its ability to uncover previously uncharacterised variation. By creating an ancestry-matched genomic reference with novel variants, we are building the essential infrastructure needed to improve diagnostic accuracy, guide therapeutic selection, and inform local healthcare policy.

Looking forward, we are in full agreement with the reviewer that establishing robust genotype-phenotype links and evaluating long-term health outcomes are paramount. These are not oversights but are integral components of the project's long-term roadmap. To this end, we are actively implementing a multidimensional outcome evaluation framework, as detailed in our roadmap paper (PMID: 40425802)[10] . This involves longitudinal surveys to track clinical utility, patient-reported outcomes, mental health impacts, and socioeconomic factors. Our unpublished preliminary data from this framework already reveal important implementation challenges and opportunities; for instance, how genomic literacy correlates with healthcare engagement and how co-occurring mental health conditions can complicate decision-making, highlighting a systemic fragmentation between physical and mental health services.

To contextualise the timeline for such outcomes, it is worth noting the trajectory of other large-scale national initiatives. The UK 100,000 Genomes Project, launched in 2013, published its first major diagnostic paper in 2021, followed by cancer and PGx outcome evaluations in 2024 and 2025 respectively. This 8-to-12-year timeline for generating and publishing comprehensive outcome data is typical for projects of this magnitude. The HKGP is on a similar path, having established the foundational dataset

that now enables the longitudinal and functional studies necessary to deliver on the full promise of genomic medicine.

Reviewer #2 Comment 2

The study suggests that population-level knowledge of genetic variants can enhance clinical diagnosis and treatment. While recognizing the importance of identifying interregional differences, **such insights do not automatically translate into better care or more effective drug therapy for individual patients**. In practice, whole-genome sequencing (WGS) is expected to become increasingly central to personalised medicine, particularly in oncology and eventually as part of routine clinical practice. Regarding genetic variants specific for tumors the WGS technique increases in application for identification of the causal variants. For these purposes, the **global distribution of variants is less relevant than the predictive power of specific genetic variants for improving drug treatment in individual cases**.

Response:

We thank the reviewer for the insightful comments and for recognising the growing importance of WGS in clinical diagnosis and personalised medicine.

We agree with the reviewer that population-level knowledge of genetic variants alone does not guarantee better care or more effective drug therapy for individual patients. To bridge this gap, our ongoing and future studies focus on integrating genomic data with electronic health records (EHR); conducting clinical trials to assess the impact of PGx-guided prescriptions; and developing AMP-like clinical guidelines tailored to local populations. This study is an initial step, in which we identified and characterised actionable genetic variants and their prevalence in the Hong Kong population. Future work will focus on implementing these findings in clinical practice; evaluating their real-world impact; and translating research insights into better care.

We also agree with the reviewer on the growing centrality of WGS for personalised medicine and clinical diagnosis. Consistent with global genome initiatives in Asia and worldwide—which demonstrate WGS's power to improve diagnostic yield and guide personalised treatment—our study further highlights its utility. We identified rare and complex variants (e.g., structural variants, CNVs, novel LoF variants in PGx genes and hereditary disorders) often missed by conventional methods.

Illustrating this in hereditary cancer (a focus of the HKGP), WGS analysis of blood DNA yielded diagnoses in 21.7% (68/315) of cases, detecting key pathogenic predisposition variants. While our study focuses on germline variation, we concur that WGS's impact in oncology will extend far beyond hereditary cancer, critically informing prognosis, tumour classification, and treatment guidance. In order to address individual variation and tumour heterogeneity effectively, the integration of multi-omics data is essential.

While the current study focuses on germline variation and its clinical implications, the HKGP actively supports projects investigating these precise tumour-focused, multi-omics applications. A relevant example is our recent HKGP glioblastoma (GB) study (PMID: 41086113)[11] . This work integrated genomic and transcriptomic profiling to identify somatic alterations such as amplified oncogenes (e.g., *EGFR, MYC, CDK4, PDGFRA, PPARGC1A*) that frequently localised on extrachromosomal DNA (ecDNA). These findings established connections between ecDNA architecture and critical aspects of GB malignancy—including malignant cell differentiation, microenvironmental remodelling, and reciprocal tumour cell-macrophage signalling—providing actionable insights for precision oncology. This study exemplifies the essential role of multi-omics approaches in deciphering tumour heterogeneity and advancing personalised oncology.

Reviewer #2 Comment 3
Relatively little emphasis is given to relevant previous studies in the field encompassing very large Chinese populations such as PMID: 40634300, PMID: 39991480 and the China Kadoorie Biobank (CKB) genetic resources.

Response:

We appreciate the reviewer highlighting these pivotal large-scale studies (PMID: 40634300, PMID: 39991480, and the China Kadoorie Biobank genetic resources)[2,3,12] . While these initiatives have significantly advanced our understanding of common variation and pharmacogenomics, they primarily rely on SNP arrays or low-depth GS, which limits the detection of rare, novel, and structural variants. In contrast, our study utilises high-depth GS to specifically address rare disease diagnosis and capture complex alleles beyond the scope of array-based approaches.

Specifically:

1. PMID: 40634300 (The Taiwan Precision Medicine Initiative) [2] is a PGx study with half a million individuals using SNP arrays and comprehensive clinical records. The study validated previous findings that PGx risk variants are significantly associated with drug-related adverse events or ineffectiveness. Its reliance on SNP arrays limits the detection of non-SNV PGx star alleles and novel variants.

2. PMID: 39991480 (PGx study from NIPT samples in China)[3] analysed 0.2 million individuals using low-depth (<0.1x) WGS and SNP arrays. While the study provided insights into PGx variants, it similarly faced limitations in detecting non-SNV variants and novel PGx alleles.

3. The China Kadoorie Biobank (CKB) is a major biobank for chronic disease research, with genetic data from 0.5 million individuals primarily derived from SNP arrays (PMID: 22158673)[12] . It has supported numerous GWAS related

studies on chronic diseases in the Chinese population, but the genetic data from SNP arrays shares limitations in detecting rare and novel variants.

We have updated the Introduction to cite these works and clarify how the HKGP complements these existing resources by filling the gap in rare variant detection.

> "*While previous initiatives have significantly advanced the understanding of common genetic variation and pharmacogenomics through arrays and low-depth genome sequencing, focusing primarily on chronic diseases and drug response, the HKGP complements these efforts by addressing the gap in RD diagnosis using genome sequencing*[21–23]" with above references. (page 7, line 178-182, in **Introduction**)

Reviewer #2 Comment 4

In the pharmacogenomic section, the authors group variants across multiple genes—such as ADME genes involved in drug response and toxicity—but provide little discussion of the relative clinical significance of the specific variants. The impact of different genetic variants on clinical outcomes varies considerably. Broader approaches, such as the European PREPARE study (>7,000 patients), did not demonstrate a reduction in drug-induced adverse reactions through predictive genotyping, largely due to the low prevalence of actionable variants, the important placebo effects and the absence of a targeted focus on specific gene–drug pairs (PMID: 40552566). In contrast, the most informative insights typically emerge from analyses of well-defined gene–drug pairs. This is underscored by the FDA's compilation of pharmacogenomic biomarkers in drug labelling, which highlights the relative importance of specific gene–drug pairs across therapeutic areas (see https://www.fda.gov/drugs/science-and-research-drugs/table-pharmacogenomic-biomarkers-drug-labeling and https://www.fda.gov/medical-devices/precision-medicine/table-pharmacogenetic-associations. Thus, the authors provide a list (Table 2) of the most commonly prescribed drugs in Hong Kong, linked to genes with actionable variants, but they do not connect this to population-specific genetic variants that could significantly influence drug response and side-effect profiles. In addition, when compared with the FDA's classification of gene–drug pairs by clinical importance in pharmacogenomics—specifically those listed in the Table of Pharmacogenetic Associations for which the Data Support Therapeutic Management Recommendations(https://www.fda.gov/medical-devices/precision-medicine/table-pharmacogenetic-associations), the overlap is limited. Of the 20 most frequently prescribed drugs in Hong Kong with actionable pharmacogenomic phenotypes (Table 2), only 3 are recognized by the FDA as having sufficient evidence to support therapeutic management recommendations.

Response:

We thank the reviewer for the thoughtful and constructive feedback regarding the pharmacogenomic section of our manuscript. We have addressed the specific concerns

by conducting additional analyses using the FDA's Table of Pharmacogenetic Associations, a valuable resource that complements CPIC guidelines by providing an evaluation of gene-drug interactions based on scientific evidence reviewed by the FDA. These analyses are now presented in **Supplementary Table 11** and described in the Methods and Results sections as follows:

> *"To further investigate the significant of the clinical impact of the actionable phenotypes (APs) in pharmacogenes, we categorised APs according to the three sections defined by the FDA Tables of Pharmacogenetic associations (www.fda.gov/medical-devices/precision-medicine/table-pharmacogenetic-associations): therapeutic management recommendations, potential impact on safety or response, and potential impact on pharmacokinetic properties. (Supplementary Table 11)."* (page 40, line 1113-1118, in **Methods**)
>
> "*Further analysis revealed an average of 2.79 out of 5.20 CPIC APs per individual were important in the U.S. Food and Drug Administration (FDA) category' supporting therapeutic management recommendations, while 1.07 APs were associated with potential impact on safety or response, and another 1.07 APs were related to potential impact on pharmacokinetic properties."* (page 21, line 530-535, **Results**)

To further address the reviewer's concern about clinical relevance, we analysed the top 50 most prescribed drugs in Hong Kong using the FDA's Table of Pharmacogenomic Biomarkers in Drug Labelling as follows:

> *"To further study the clinical relevance, we analysed these prescribed drugs using the FDA's Table of Pharmacogenomic Biomarkers in Drug Labeling (www.fda.gov/drugs/science-and-research-drugs/table-pharmacogenomic-biomarkers-drug-labeling), and identify clinically consequential pharmacogenomic information with three key labelling sections: adverse reactions, warnings and precautions, and dosage and administration."* (page 41, line 1138-1144, in **Methods**)
>
> "Expanding to the top 50, we observed that 13 drugs carried FDA-recognised gene-drug associations with therapeutic management recommendations, and 16 had clinically important labels (i.e., adverse reactions, warnings and precautions, or dosage and administration)." (page 22, line 555-558, in **Results**; and more details in **Supplementary Table 12**)
>
> "*While only 3 of the 20 most prescribed drugs (and 13 of the top 50) carry FDA-recognised gene-drug associations with therapeutic management recommendations, and 16 of the top 50 drugs carry FDA clinically important labels, this regulatory threshold represents a floor—not a ceiling—for clinically actionable pharmacogenomics. it highlights an urgent need for translational*

frameworks that bridge biomarker discovery to bedside application." (page 30, line 773-778, in **Discussion**)

These additional analyses and discussions on FDA PGx resources, together with original content based on AMP and CPIC, provide a more comprehensive evaluation on the clinical importance of pharmacogenomic associations evaluated in our study. We hope these revisions address the reviewer's concerns and strengthen the manuscript.

Reviewer #2 Comment 5

Since all individuals were recruited based on suspicion of having a genetic disease, it is not appropriate to consider them as a general disease cohort. They are clearly distinct from the broader population with respect to germline variation.

Response

We thank the reviewer for this important observation regarding the recruitment of the HKGP. To address potential biases while maintaining scientific validity, we implemented a multi-faceted approach. First, we deliberately included a distinct subcohort of 10,599 unaffected individuals, who were not under investigation for any genetic condition at recruitment, in our HKGP Chinese cohort. This group underwent stringent filtering for unrelatedness and Southern Chinese ancestry, serving as the exclusive basis for all population-level analyses in this study, including clinically actionable findings and PGx landscape.

Second, to avoid biased representation in the analysis of the 7,662 affected individuals in HKGP Chinese cohort, we implemented rigorous condition-specific filtering to minimise overrepresentation of pathogenic variants. Probands whose primary clinical indication matched analysed ACMG dominant genes were excluded from relevant analyses. For recessive gene analyses, we excluded individuals with children affected by diseases matching carrier screening genes. Similarly, for PGx genes associated with congenital conditions, individuals with matching phenotypes (either personal or in offspring) were excluded as detailed in our response to Comment 6. These measures ensured variant frequency analyses weren't skewed by clinical ascertainment bias.

We acknowledge that the HKGP cohort is distinct from the broader general population due to the inclusion of symptomatic individuals and their families, but the dual design enables clinically actionable genetic discoveries while providing controlled population references for Hong Kong Chinese.

For transparency, we have added a breakdown of the participants in each group in the **Supplementary Information** (page 2, line 55-70).

Number of individuals for each group (completed sequencing, variant calling and classification by Nov 2024, n = 24,112)

1) *Undiagnosed Disorders and Hereditary Cancers (all affected)*

   a) *10,747 were sequenced.*
   b) *2,227 completed curation and were included in the diagnostic cohort.*
   c) *5,568 Chinese singletons were included in the HKGP Chinese cohort.*
2) *Genomics and Precision Health cohorts (affected and unaffected)*
   a) *5,819 were sequenced.*
   b) *No participant was included in the diagnostic cohort*
   c) *5,794 Chinese were included in the HKGP Chinese cohort, including 3,700 unaffected and 2,094 affected by chronic diseases*
3) *Unaffected first-degree family members*
   a) *7,546 were sequenced.*
   b) *2,378 family members of the curated probands in the diagnostic cohort,*
   c) *6,899 Chinese were included in the HKGP Chinese cohort (non-Chinese or related members of the same family unit were excluded).*

We appreciate this opportunity to clarify the recruitment strategy, cohort composition, and analytical approach. We have carefully reviewed the **Supplementary Information** to ensure these distinctions are explicitly stated.

Reviewer #2 Comment 6

Some pharmacogenes have endogenous substrates (e.g., ABCG2) and are linked to congenital diseases. The cohort may therefore be enriched for such mutations, and this potential bias should be considered in the pharmacogenomic profiling.

Response:

Thank you for your insightful comment. We fully acknowledge the potential bias in PGx allele frequencies if congenital disease-associated pharmacogenes are enriched in our cohort. To address this, we excluded individuals from the HKGP Chinese cohort if either their own or their offspring's primary indication matched diseases linked to seven pharmacogenes with established gene-disease validity. This exclusion, detailed in the Methods, removed four affected individuals (one with haemolytic anaemia due to G6PD deficiency, one with RYR1 myopathy, and two with hearing loss due to MT-RNR1 variants), resulting in 18,257 individuals for unbiased PGx analysis:

> *"Among the 25 pharmacogenes analysed, seven pharmacogenes (CACNA1S, CFTR, DPYD, G6PD, MT-RNR1, RYR1, VKORC1) are associated with congenital diseases as classified by ClinGen with definitive, strong, or moderate gene-disease validity, or as Green (diagnostic) or Amber (borderline) in relevant disease panels in Genomics England PanelApp and PanelApp Australia (similar gene selection approach for the diagnostic cohort). To avoid confounding effects from these conditions, individuals from the HKGP Chinese cohort were excluded from the analysis if their own or their offsprings' primary phenotypes matched the associated congenital diseases. The remaining individuals were included for the*

pharmacogenomic analysis of known alleles and novel variants. This approach was taken to ensure that the analysis focused on unbiased pharmacogenomic findings unrelated to pre-existing diagnoses linked to these specific pharmacogenes" (page 39, line 1087-1098, in **Methods**)

"To prevent analysis bias, we excluded four individuals with personal or offspring phenotypes linked to seven congenital disease-associated pharmacogenes (classified by ClinGen and panelApp; see methods) of the final cohort for pharmacogenomic profiling comprised 18,257 individuals." (page 20, line 495-498, in **Results**, section)

Regarding *ABCG2*, it is not associated with congenital diseases in ClinGen, Red (low level of evidence) in PanelApp and no pathogenic/likely pathogenic variants (except large CNVs) are reported in ClinVar. *ABCG2* variants are linked to urate transport disorders (hyperuricemia and early-onset gout) via genome-wide studies (PMID: 34206432)[13] . The allele frequency in HKGP of the key PGx variant rs2231142 (0.32) in *ABCG2* aligns with the East Asian population frequency (0.31 per CPIC), indicating no bias (**Supplementary Tables 9 and 11**).

This approach ensures our PGx findings are not confounded by pre-existing diagnoses. Importantly, excluded individuals may still benefit from genetic counselling regarding these pharmacogenes for their clinical management.

Reviewer #2 Comment 7

Figure 1a could be reordered—by either sample size or diagnostic yield—to present the message more clearly.

Response

Thank you for the suggestion. We agree that reordering Figure 1a would help present the message more clearly. As such, we have reordered the disease categories by sample size, which we believe provides a better visual representation of the data.

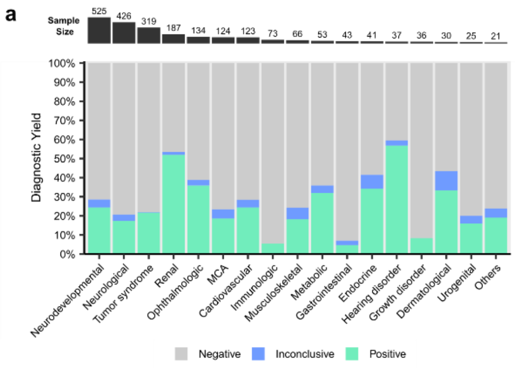

Reviewer #2 Comment 8

In Figure 4a, it appears that each gene was compared to a single CPIC reference population. How was this reference population selected?

Response

Thank you for your question. In Figure 4a, the "reference population" was selected as the CPIC population with the largest sample size for each gene. This approach ensures the most robust and stable estimates of allele frequency for comparisons, while avoiding the arbitrary selection of one group as the standard for comparison. We recognise that "reference population" is not an official term from CPIC. To prevent any misunderstanding, we have updated the manuscript and figure to use the term "CPIC max population" for each gene.

> "reference population" was replaced by "*CPIC population with max sample size (max population).*" (page 20, line 501-502, in multiple sentences in **Results** and **Figure 4a** and its legend)

This revised terminology better reflects the methodology used and aligns with similar approaches reported in other publications.

Reviewer #2 Comment 9

In Table 1, the first category should be the 553 patients with positive genetic diagnoses, rather than the entire diagnostic cohort.

Response

Thank you for your suggestion. You are correct that presenting the 553 patients with positive genetic diagnoses as the first category provides greater clarity. We have updated Table 1 accordingly and revised the related sentences in the Results section to reflect this change.

> *"Diagnostic cohort (Probands with positive finding, n=553)"* in **Table 1**

Reviewer #4 Comment 1

Were results returned to all participants, and if so how? I recognized that the rare disease diagnoses were likely returned to the clinicians at the respective hospitals, but what about the carrier screening outcomes for example. How was education of participants supported so they could understand their results? Can you add more details or a section addressing this in the methods as returning this number of actionable results is no minor undertaking.

Response

Thank you for the comment. We have expanded the section to address findings disclosure, particularly noting:

> *"Three years post-pilot, HKGP stands at a critical juncture in genomic findings disclosure. While our current protocol returns only primary findings, it was developed with careful consideration of key stakeholders' preferences[35], and this study serves as the first step toward broader return options."* (page 27, 657-661, in **Discussion**)

We have also added the details on reporting and returning results in the **Supplementary Information** as follows:

> ***Primary Findings***
> *Building on patient and clinician feedback, we will continue to prioritise returning clinically significant findings directly related to the referral indication and clinical phenotype.*
>
> ***Additional medically actionable findings***
> ***Dominant Disorders:*** *For participants opt-in for feedback of additional findings of GS, we have developed a plan for reporting and returning findings in 13 genes (of which, 12 are associated with dominant disorders) – MLH1; MSH2; MSH6; MUTYH; APC; BRCA1; BRCA2; VHL; MEN1; RET; LDLR; APOB; PCSK9 – based on clinical actionability and severity. The gene list will be reviewed and revised regularly, by the Scientific and Ethics Advisory Committees of HKGI, to align with evolving international standards. In compliance with ACMG guidelines and reporting guidance, only pathogenic or likely pathogenic variants will be reported* (https://search.clinicalgenome.org/kb/genes/acmgsf). *Specific conditions apply: for child participants, only genes associated with childhood-onset diseases will be returned. This structured approach ensures responsible return of high-impact genetic information while respecting clinical context and participant preferences.*
>
> ***Recessive Disorders:*** *For reporting and returning additional findings of MUTYH-associated polyposis, only individuals with two identified disease-causing variants will receive results. Regarding expanded carrier screening, we are at the crossroads. While we will continue to return carrier status upon patient request,*

> *this study reinforces our decision to develop a Chinese-specific carrier screening panel rather than relying solely on resources based on European ancestries like ACMG and Mackenzie's Mission. We have demonstrated our capability to identify and return these results to patients.*

To ensure participants understand the project and their results, we implemented a comprehensive education and counselling framework centred on transparency and participant empowerment, as identified in our focus group studies (PMID: 37600657)[14] . Clinical geneticists and genetic counsellors served as the critical communication bridge between clinicians and participants. They provided clear explanations of the medical, psychosocial, and familial implications of genetic findings, while offering psychosocial support to help participants adapt and adjust. These professionals were equipped to address social, ethical, and legal aspects, ensuring informed decisions aligned with principles of autonomy, non-maleficence, and beneficence. In addition, participants received tailored multimedia resources—including two versions of information booklets and 11 educational videos for adults and teenagers—that explained project details, benefits, risks, and rights. During pre- and post-test sessions, clinical geneticists and genetic counsellors used these resources to facilitate interactive discussions, ensuring all participant questions and concerns were addressed. Based on participant feedback, 84.6% agreed the counselling sessions effectively prepared them to make informed decisions about their health (PMID: 40188098)[15] .

Reviewer #4 Comment 2

Similarly, how did you deal with clinically actionable findings that were not related to the referral indication for children and youth - so the 564 dominant disorder genes and the ACMG-listed recessive disorder genes – were these returned even if not actionable in childhood? Was there a choice about being informed about these risks for those under 18 years of age?

Response

Thank you for the comment. Consistent with our framework for returning additional medically actionable findings (as in the response above), we have implemented stringent condition-specific reporting protocols, e.g.,

> *"for child participants, only genes associated with childhood-onset diseases will be returned."* (page 3, line 87, in **Supplementary Information**)

While minor participants still rely on proxy consent by parents/legal guardians and/or child assent, Child participants will be recontacted upon reaching adulthood to independently decide on continued participation or withdrawal, including their preferences for receiving additional medically actionable findings. This best-practice reconsent approach follows established protocols from international genome projects.

Reviewer #4 Comment 3

Can you comment on the use of buccal/saliva? How many of the samples were buccal/saliva, what was the quality? In my experience, buccal samples for genomes tend to be messy and require much higher depth of coverage, especially for the identification of de novo variants.

Response

Thank you for raising this point. Only 2.5% of the samples in our study were collected from buccal/saliva. As you correctly noted, the quality of these samples, particularly saliva samples, tends to be lower compared to blood samples. In our dataset, relatively lower depth of coverage was observed in saliva samples comparing with blood samples (37.0x vs 39.0x). However, we only retained samples with depth exceeding the threshold (>29.5x), which is the same threshold applied to all samples. Further evaluation on the unmappable reads revealed they were mostly contributed by oral bacteria.

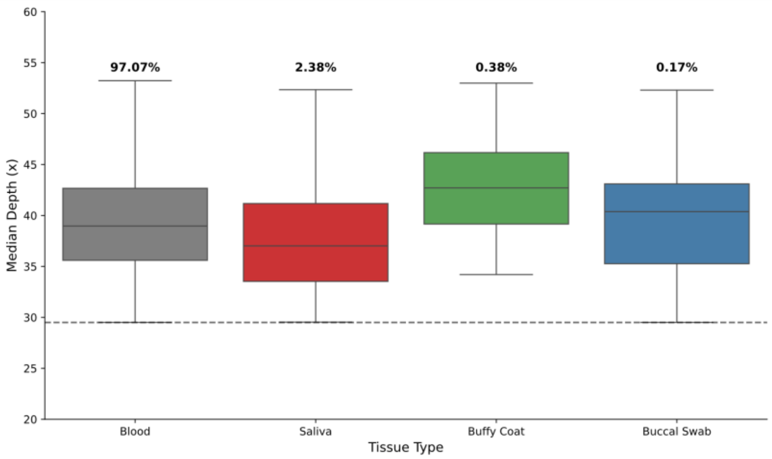

Other QC parameters, such as mapping ratio, fraction of genome passing 10x, and fraction of genes passing 15x, were comparable between buccal/saliva and blood samples. A detailed comparison of QC metrics by sample type has summarised below.

Sample type	***Median Depth [interquartile range]***	***Genome 10x Ratio [S.D.]***	***PCT Mapping Rate [S.D.]***	***PCT Gene Passed 15x Ratio [S.D.]***
Blood	*39.0 [35.6-42.7]*	*0.956 ± 0.00495*	*0.999 ± 0.000531*	*0.992 ± 0.00493*
Saliva	*37.0 [33.5-41.2]*	*0.952 ± 0.00507*	*0.998 ± 0.00143*	*0.992 ± 0.00541*
Buffy Coat	*42.7 [39.2-46.2]*	*0.956 ± 0.00481*	*0.999 ± 0.000191*	*0.994 ± 0.00249*
Buccal Swab	*40.4 [35.3-43.1]*	*0.956 ± 0.00504*	*0.998 ± 0.00389*	*0.992 ± 0.00727*
All	*38.9 [35.6-42.7]*	*0.956 ± 0.00496*	*0.999 ± 0.000617*	*0.992 ± 0.00495*

For individual variant quality, we seldom observed false-positive de novo variants, mainly insertions in buccal/saliva samples. To address this, all pathogenic/likely

pathogenic (P/LP) variants in the diagnostic cohort, as well as some novel insertions identified in the Chinese cohort, were manually validated using IGV.

In light of these observations, we are reducing the collection of non-blood samples in the main phase of the project. Buccal/saliva samples will only be collected for participants where blood collection is challenging due to health conditions.

Reviewer #4 Comment 4

On line 868, it states that orthogonal validation was performed for P/LP diagnostic findings – can you expand on this in the methods? Does that mean that all SNVs, CNVs, STRs etc were confirmed in a secondary assay? With a new sample from the participant? In particular, in my experience bioinformatic tools such as Expansion Hunter aren't yet reliable in calling STRs, especially with short read data, so secondary testing would be important to confirm expansion size.

Response

Thank you for your question and for highlighting the importance of orthogonal validation for different types of variants. We have clarified the methods section to provide additional details regarding the validation process:

> *"Orthogonal validation was performed for all P/LP variants using independent DNA extracted from the original sample. For SNVs and indels, Sanger sequencing was used, while SVs, STR expansions, and mosaic SNVs/indels were validated using Nanopore long-read sequencing."* (page 35, line 936 – 939, in **Methods**)

We agree that STR calls, particularly with tools like Expansion Hunter using short-read data, have a high false positive rate. This is why we used long-read sequencing for STR validation, as it achieves better accuracy in resolving these regions and confirming expansion size.

Reviewer #4 Comment 5

On line 870, it states that VUS were reported only if they were clinically actionable. Does this mean that only if a secondary assay, like enzyme or biomarker testing, could confirm the diagnosis? Please expand.

Response

Thank you for your comment. To clarify, "clinically actionable" in this context refers to:

> *"VUS that meet following criteria agreed upon by all parties in the multidisciplinary team (MDT), including clinicians: highly compatible with the clinical phenotypes, and when additional secondary assay/analysis—such as RNA sequencing, enzyme activity testing, immunohistochemical staining, imaging*

studies, and segregation analysis, can be performed to confirm the diagnosis." (page 35, line 940-945, in **Methods**)

Reviewer #4 Comment 6

How is the HKGP contributing to global datasets like gnomAD and ClinVar so that all Asian individuals can benefit no matter where they live in the world?

Response:

HKGI has engaged with leaders from various genome institutes and genome projects, including the Hospital for Sick Children (Canada), Harvard Medical School (USA), Australian Genomics, Genomics England and other universities, to explore the clinical, personal, and system-wide value of genomic medicine. We contributed to

1. Defining utility metrics: Advancing tools like the GUV Scale (Genomic Utility Value) for policy decisions (PMID: 41310283)[9]
2. Global data sharing: Supporting GA4GH standards to scale genomic data interoperability across health systems.

HKGP has a strategic plan in place to contribute to global datasets such as ClinVar, ensuring that Asian individuals can benefit from these resources regardless of where they live. Specifically:

1. Contribution Strategy: Variants classified as pathogenic or likely pathogenic are released to clinicians following diagnostic reporting. Updates or additional feedback on the pathogenicity of these variants will be incorporated within 6 months. After this process, the variants will be summarised for publication and submitted to ClinVar to ensure they are shared with the broader scientific and clinical community.
2. Progress to Date: As part of this strategy, the first batch of 45 variants identified in our pilot study, published earlier this year and also included in the diagnostic cohort in this study, has already been uploaded to ClinVar. (Submission ID: SUB15757748; Submitter: Hong Kong Genome Institute https://www.ncbi.nlm.nih.gov/clinvar/submitters/510250/)
3. Future Contributions: For the diagnostic cohort, which includes 573 variants, we plan to follow the same protocol. These variants, as well as other newly curated positive variants beyond this study, will be uploaded to ClinVar or other related public repositories in batches, ensuring timely sharing of valuable genomic insights.

This approach underscores HKGP's commitment to advancing the representation of Asian populations in global genomic datasets and fostering equitable access to genomic

knowledge worldwide. **Supplementary Table 2** was updated by adding the ClinVar submitter URL.

Reviewer #4 Comment 7

The 17.5% of diagnosed patients who became clinical trial eligible seems high (Figure 1). Can you expand on what types of trials they were eligible for as my review of the diagnostic details suggests there are many different diseases captured in that percentage.

Response:

We thank the reviewer for highlighting the need for further clarification regarding the clinical trial eligibility of the 17.5% diagnosed patients. Clinical trial eligibility in our study was defined as follows and added to Method:

> "*Meet enrolment criteria for phase II or higher interventional (related to drugs, medical devices, procedures, vaccines as defined in clinicaltrials.gov) or observational (focused on assessing non-interventional biomedical or health outcomes) trial studies listed in clinicaltrials.gov or clinicaltrialsregister.eu that were related to the patient's target gene and disease at the time of diagnosis.*" (page 37-38, line 1029-1033, **Methods**)

Among the subgroup of 97 patients eligible for clinical trials, the major diseases are as follows: *GJB2*-related nonsyndromic hearing loss and deafness (n=22) and *NF1*-related neurofibromatosis type 1 (n=28). The number will reduce to 75 (13.6%) if we only include interventional trials.

> *"Among the 97 probands eligible to clinical trials (17.5%), 75 (13.6%) were eligible for interventional clinical trials, with 9 of these involving phase III/IV trials, while 22 (3.9%) were eligible for observational clinical trials."* (page 10, line 268-270, in **Results**)

We hope this additional information addresses the reviewer's concerns about the diversity of diseases and trial types represented in the eligible cohort.

Reviewer #4 Comment 8

While high altered-function allele frequencies—such as those exceeding 10% for ABCG2, CYP2B6, UGT1A1, HLA-B, SLCO1B1, and CYP2D6—may suggest population-level relevance, AMP's criteria for reportable pharmacogenes are based on clinical actionability, not frequency alone. This would be important to keep in mind because how would a region tailor pharmaceutical dosing for 0.9M people; definitely challenges with scaling here.

Response

We fully acknowledge that AMP's criteria prioritise three key elements: clinical actionability, allele frequency, and the availability of reference materials. While we highlight allele frequency as an important factor in our manuscript, we agree it is not the sole determinant of clinical utility. Our analysis integrates frequency data with clinical evidence to provide a broader perspective. Notably, two genes in question—*CYP2D6* and *HLA-B*—are associated with therapeutic management recommendations on the FDA PGx Association list, demonstrating their high clinical actionability. Specifically, *HLA-B* *15:02 (AF=0.08) and *58:01 (AF=0.07) guide carbamazepine and allopurinol prescribing through pre-emptive pharmacogenomic testing in the Hospital Authority of Hong Kong. Given their population prevalence in Hong Kong and technological advances (e.g., WGS enabling reliable CYP2D6 genotyping), these genes warrant expanded consideration in AMP guidelines. To support this, **Supplementary table 11** now includes FDA's PGx Association for each gene and phenotypes/alleles. **Supplementary table 12** lists top 50 mostly prescribed drugs in Hong Kong with FDA's drug labels on adverse reactions, warnings and precautions, and dosage and administration.

Reviewer #4 Comment 9

Line 182: 'Demographics and clinical characteristics OF the diagnostic cohort'

Response

Updated from 'for' to 'of' in **Extended Data Table 1**.

Reviewer #4 Comment 10

Fig 1b, please indicate that the total number who received a diagnosis was 553

Response:

Thank you for your suggestion. We have added a text box to **Figure 1.b** with the term "Positive Diagnosis n=553." The design is clear and ensures that readers can easily compare the total number of probands with a diagnosis and their corresponding types of clinical management.

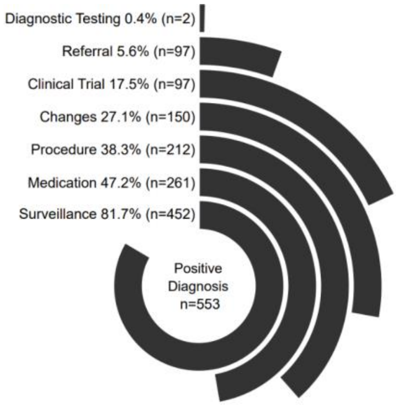

Reviewer #4 Comment 10

Suppl Table 9 – 'DecreasEd metabolism of nicotine'

Response

Typo fixed in **Supplementary Table 9**.

References

1. Yang, H.-C. *et al.* The Taiwan Precision Medicine Initiative provides a cohort for large-scale studies. *Nature* 1–11 (2025) doi:10.1038/s41586-025-09680-x.

2. Wei, C.-Y. *et al.* Clinical impact of pharmacogenetic risk variants in a large chinese cohort. *Nat. Commun.* **16**, 6344 (2025).

3. Wang, L.-Y. *et al.* The pharmacogenomic landscape in the Chinese: An analytics of pharmacogenetic variants in 206,640 individuals. *Innov.* **6**, 100773 (2025).

4. Walters, R. G. *et al.* Genotyping and population characteristics of the China Kadoorie Biobank. *Cell Genom.* **3**, 100361 (2023).

5. Stark, Z. *et al.* Australian Genomics: Outcomes of a 5-year national program to accelerate the integration of genomics in healthcare. *Am. J. Hum. Genet.* **110**, 419–426 (2023).

6. Sosinsky, A. *et al.* Insights for precision oncology from the integration of genomic and clinical data of 13,880 tumors from the 100,000 Genomes Cancer Programme. *Nat. Med.* **30**, 279–289 (2024).

7. Leong, I. U. S. *et al.* Large-Scale Pharmacogenomics Analysis of Patients With Cancer Within the 100,000 Genomes Project Combining Whole-Genome Sequencing and Medical Records to Inform Clinical Practice. *J. Clin. Oncol.* **43**, 682–693 (2025).

8. Smedley, D. *et al.* 100,000 Genomes Pilot on Rare-Disease Diagnosis in Health Care — Preliminary Report. *N. Engl. J. Med.* **385**, 1868–1880 (2021).

9. Goranitis, I. *et al.* Determining the value of genomics in healthcare. *Nat. Med.* 1–12 (2025) doi:10.1038/s41591-025-04061-3.

10. Chung, C. C. Y., Chu, A. T. W. & Chung, B. H. Y. A roadmap for genome projects to foster psychosocial and economic evidence to further policy and practice. *Commun. Med.* **5**, 198 (2025).

11. Tang, W. *et al.* Integrated genomic and transcriptomic profiling of glioblastoma reveals ecDNA-driven heterogeneity and microenvironmental reprogramming. *Cell Rep.* **44**, 116426 (2025).

12. Chen, Z. *et al.* China Kadoorie Biobank of 0.5 million people: survey methods, baseline characteristics and long-term follow-up. *Int. J. Epidemiology* **40**, 1652–1666 (2011).

13. Eckenstaler, R. & Benndorf, R. A. The Role of ABCG2 in the Pathogenesis of Primary Hyperuricemia and Gout—An Update. *Int. J. Mol. Sci.* **22**, 6678 (2021).

14. Chu, A. T. W., Chung, C. C. Y., Hue, S. P. Y. & Chung, B. H. Y. The growing needs of genetic counselling—Feasibility in utilization of tele-genetic counselling in Asia and Hong Kong. *Front. Genet.* **14**, 1239817 (2023).

15. Chu, A. T. *et al.* Experiences of participants with undiagnosed diseases and hereditary cancers during the initial phase of the Hong Kong genome project: a mixed-methods study. *Hum. Genom.* **19**, 36 (2025).

Reviewers' Comments:

Reviewer #1 (Remarks to the Author):
Thank you for the opportunity to review a revised version of this manuscript.
All of my previous comments have been addressed well.

Reviewer #1 Comment 1: I would still strongly encourage the authors to make a large submission to ClinVar as part of this publication, even if it is not for all the variants.
Response: We appreciate the reviewer's encouragement to submit variants to ClinVar. We are actively submitting variants from the diagnostic cohort of the HKGP to ClinVar, aligned with the progress of collecting clinician feedback on GS-based diagnoses. As of January 2026, we have submitted multiple batches, totalling over 140 variants. This submission process is ongoing, and we are committed to submitting all (or most) of the variants from the diagnostic cohort over time. The submitter summary link has been included in the "**Data Availability**" section of the manuscript.

> *"Variants identified in the diagnostic cohort were uploaded to ClinVar in batches (https://www.ncbi.nlm.nih.gov/clinvar/submitters/510250/)."*

Reviewer #2 (Remarks to the Author):
Reviewer 2 Comments to rebuttal

Reviewer #2 Comment 1
In the Hong Kong Genome Project (HKGP) over 20,000 individuals were sequenced, combining a rare disease cohort with a broader Chinese sample. It reported a 25% diagnostic yield in rare disease patients and developed ancestry-matched references that highlighted shortcomings of European-centric screening panels. While these contributions are important, the project's impact is constrained by its narrow geographical scope. Unlike large-scale initiatives such as the UK Biobank or the U.S. All of Us program, which integrate genomic data with extensive longitudinal and phenotypic records, the HKGP offers little insight into long-term health outcomes or broader population-level trends. The pharmacogenomic analysis reveals Hong Kong-specific alterations in the distribution of different genetic variants where such differences with

respect to interethnic variations have been extensively published in previous studies and databases. With respect to other cohorts novel Chinese-specific variants were identified without any important data on altered phenotypes or risks, merely interethnic differences in the distribution of P/LP variants. As a result, the HKGP represents a valuable starting point for Hong Kong-specific genomic resources but falls short of delivering novel holistic genotype–phenotype links and global comparability achieved by other major international efforts.

Reviewers new response
In the authors' response, no new information is provided that would alter the original statement that this study is geographically restricted and lacks novel holistic genotype–phenotype links and that this kind of investigation have previously done in major international projects.
Response:

We appreciate the reviewer's comments and recognise the critique regarding the geographical scope and the absence of holistic genotype–phenotype links in our study. However, as the editor has thoughtfully pointed out, addressing these aspects falls beyond the scope of the present work. The primary objective of our study is to establish a foundation for Hong Kong-specific genomic resources and to highlight population-level differences, rather than to replicate the global comparability or longitudinal insights achieved by larger international initiatives. This limitation is inherent to the design and scope of our current study, but will be covered by other studies under HKGP in the future. Unfortunately, we were unable to discuss this limitation in detail in the manuscript due to word-limit constraints.

Reviewer #2 Comment 2
The study suggests that population-level knowledge of genetic variants can enhance clinical diagnosis and treatment. While recognizing the importance of identifying interregional differences, such insights do not automatically translate into better care or more effective drug therapy for individual patients. In practice, whole-genome sequencing (WGS) is expected to become increasingly central to personalised medicine, particularly in oncology and eventually as part of routine clinical practice. Regarding genetic variants specific for tumors the WGS technique increases in application for identification of the causal variants. For these purposes, the global distribution of variants is less relevant than the predictive power of specific genetic variants for improving drug treatment in individual cases.

Reviewers new response

A summary of the reviewers statement here is that the reviewer is sceptical that population- or geography-based variant data meaningfully improves individual patient care and argues that clinically actionable, predictive genetic information obtained through WGS is more important than knowing how variants are distributed across populations. This criticism is still valid

Response:

We thank the reviewer for their thoughtful comment. We acknowledge that the predictive power of clinically actionable genetic variants obtained through whole-genome sequencing plays a critical role in advancing personalised medicine. However, as the editor has thoughtfully pointed out, addressing these aspects falls beyond the scope of the present study. Our current work focuses on the population-level distribution of genetic variants and its potential to inform clinical practice on a broader scale, while our future studies will cover its translation of clinical actionability, as outlined in the discussion of the revised manuscript.

> *"Additional work, such as clinical trials like the PREPARE study, is needed to link population-specific variants to clinically meaningful drug response or adverse-effect profiles (e.g., effect sizes, penetrance, and outcomes in real-world care) before they can be considered for guideline implementation."*

Reviewer #2 Comment 3
Relatively little emphasis is given to relevant previous studies in the field encompassing very large Chinese populations such as PMID: 40634300, PMID: 39991480 and the China Kadoorie Biobank (CKB) genetic resources.
Reviewers new response
In contrast to the authors' response, the reviewer considers these large studies to be highly important and believes they should be discussed in the manuscript, including differences in methodology. However, the authors have chosen not to address them.

Response:

We have updated the discussion by addressing the importance of existing precision medicine initiatives for Chinese and how HKGP complements these efforts and

compared the difference of the methodology and focused disease areas among the projects. (Discussion line 444-451)

> *"Previous Chinese precision medicine initiatives, including Taiwan Precision Medicine Initiative, China Kadoorie Biobank, and PGx studies in China, have advanced our understanding of common genetic variation and pharmacogenomics, primarily focusing on chronic diseases and drug response using SNP arrays or low-depth GS. The HKGP complements these efforts by employing high-depth GS, enabling the study of rare diseases and the identification of novel, complex and structural variants. Together, these initiatives play a vital role in building a comprehensive foundation for precision medicine, with HKGP addressing an important gap by focusing on RD."*

Reviewer #2 Comment 4

In the pharmacogenomic section, the authors group variants across multiple genes such as ADME genes involved in drug response and toxicity but provide little discussion of the relative clinical significance of the specific variants. The impact of different genetic variants on clinical outcomes varies considerably. Broader approaches, such as the European PREPARE study (>7,000 patients), did not demonstrate a reduction in drug induced adverse reactions through predictive genotyping, largely due to the low prevalence of actionable variants, the important placebo effects and the absence of a targeted focus on specific gene–drug pairs (PMID: 40552566). In contrast, the most informative insights typically emerge from analyses of well-defined gene–drug pairs. This is underscored by the FDA's compilation of pharmacogenomic biomarkers in drug labelling, which highlights the relative importance of specific gene–drug pairs across therapeutic areas (see https://www.fda.gov/drugs/science-and-research-drugs/tablepharmacogenomic-biomarkers-drug-labeling and https://www.fda.gov/medicaldevices/precision-medicine/table-pharmacogenetic-associations. Thus, the authors provide a list (Table 2) of the most commonly prescribed drugs in Hong Kong, linked to genes with actionable variants, but they do not connect this to population-specific genetic variants that could significantly influence drug response and side-effect profiles. In addition, when compared with the FDA's classification of gene–drug pairs by clinical importance in pharmacogenomics—specifically those listed in the Table of Pharmacogenetic Associations for which the Data Support Therapeutic Management Recommendations(https://www.fda.gov/medical-devices/precision-

medicine/tablepharmacogenetic-associations), the overlap is limited. Of the 20 most frequently
prescribed drugs in Hong Kong with actionable pharmacogenomic phenotypes (Table 2), only 3 are recognized by the FDA as having sufficient evidence to support therapeutic management recommendations.

Reviewers new response
In summary, the reviewer argues that the pharmacogenomic analysis is overly broad and insufficiently grounded in clinically validated gene–drug relationships. The reviewer thinks that the study overstates the clinical relevance of its findings. Although the authors present a list of commonly prescribed drugs in Hong Kong linked to genes with actionable variants, they fail to connect these drugs to population-specific genetic variants that could meaningfully influence drug response or adverse-effect profiles. Consequently, the reviewer concludes that the analysis lacks population-specific actionable insights and does not align well with established regulatory evidence for pharmacogenomic utility.

Response:
Thank you for your insightful feedback. We acknowledge your concern regarding the need to connect population-specific genetic variants with clinically meaningful drug response or adverse-effect profiles. In response, we have tempered our statements on the immediate clinical relevance of the findings and added the following to the **Result** (line 376, 399-401) and **Discussion** (line 540-544):

> *"Their frequencies suggest the need for further investigation to determine their potential for reporting under AMP guidelines"*
>
> *"These findings highlight the opportunity to enhance prescribing practices, and improve clinical care, with further research to substantiate their clinical utility."*
>
> *"Additional work, such as clinical trials like the PREPARE study, is needed to link population-specific variants to clinically meaningful drug response or adverse-effect profiles (e.g., effect sizes, penetrance, and outcomes in real-world care) before they can be considered for guideline implementation."*

This clarification helps readers understand the clinical significance of the study while acknowledging its current limitations.

Reviewer #2 Comment 5
Since all individuals were recruited based on suspicion of having a genetic disease, it is not appropriate to consider them as a general disease cohort. They are clearly distinct from the broader population with respect to germline variation.

Reviewers new response:
The authors have now clarified the recruitment strategy, cohort composition, and analytical approach.

Reviewer #2 Comment 6
Some pharmacogenes have endogenous substrates (e.g., ABCG2) and are linked to congenital diseases. The cohort may therefore be enriched for such mutations, and this potential bias should be considered in the pharmacogenomic profiling.

Reviewers new comments
The authors have satisfactory dealt with this point.

Reviewer #2 Comment 7
Figure 1a could be reordered—by either sample size or diagnostic yield—to present the message more clearly.

Reviewers new comment: This has been satisfactory dealt with
.
Reviewer #2 Comment 8
In Figure 4a, it appears that each gene was compared to a single CPIC reference population. How was this reference population selected?

Reviewers new comment: This has been satisfactory dealt with

Reviewer #2 Comment 9
In Table 1, the first category should be the 553 patients with positive genetic diagnoses, rather than the entire diagnostic cohort.

Reviewers new comment: This has been satisfactory dealt with

Reviewer #4 (Remarks to the Author):

Thank you to the authors for their thoughtful responses to my previous comments. I appreciated the data availability update; from your responses, it looks like you will not be contributing the aggregate allele frequency data to gnomad at this time but will submit the P/LP variants to ClinVar. I also appreciated the clarity on which/how results were being returned at this stage of the program and recognize that the program needs to understand the scale of the challenge before secondary findings, carrier results, and pharmacogenomics are returned at scale.

Minor comments:

Line 939: edits to: "VUSs in dominant genes that meet following criteria, agreed upon by all parties in the multidisciplinary team (MDT), including clinicians, were reported: highly compatible with the clinical phenotypes, and when additional secondary assay/analysis—such as RNA sequencing, enzyme activity testing, immunohistochemical staining, imaging studies, and segregation analysis, can be performed to confirm the diagnosis."
Response: We have updated Methods according to reviewer's suggestion. (Methods line 730-735)

Line 267: re: "Among the 97 probands eligible to clinical trials (17.5%), 75 (13.6%) were eligible for interventional clinical trials, with 9 of these involving phase III/IV trials, while 22 (3.9%) were eligible for observational clinical trials." GJB2-related hearing loss and NF1 account for 50 patients of the 97 – please add this detail so that no one thinks it is 97 different trials.
Response: We have updated the details of the eligible clinical trials and relocated them to the Figure 1 legend to better manage the word count in the main text:

> *"Among the 97 probands eligible to clinical trials (17.5%), 75 (13.6%) were eligible for interventional clinical trials, with 9 of these involving phase III/IV trials, while 22 were only eligible for observational clinical trials. Notably, GJB2-related hearing loss and NF1 account for 50 of the 97 probands."*

This section needs an edit for repeating words/phrases (page 6, line 164-175, in Introduction) – see suggestions here:

“The integrated results derived from the project’s data involve four key components and pursue four core aims: (1) characterising population-wide diagnostic genomic variation in Hong Kong; (2) enabling early intervention and preventive care for asymptomatic individuals through an analysis of pathogenic or likely pathogenic (P/LP) variants in genes associated with dominant disorders, such as tumour syndromes and cardiovascular diseases; (3) informing reproductive planning through an analysis of carrier frequencies for recessive genetic disorders; and (4) optimising treatment efficacy, reducing adverse drug reactions, and improving therapeutic outcomes through pharmacogenomic profiling.”

Response: We have updated the core aims in the introduction according to reviewer’s suggestion. (Introduction line 137-144)
